# Inhibition of the upregulated phosphodiesterase 4D isoforms improves SERCA2a function in diabetic cardiomyopathy

**DOI:** 10.1111/bph.17411

**Published:** 2024-12-11

**Authors:** Zhenduo Zhu, Qiuyun Guan, Bing Xu, Sherif Bahriz, Ao Shen, Toni M. West, Yu Zhang, Bingqing Deng, Wei Wei, Yongsheng Han, Qingtong Wang, Yang K. Xiang

**Affiliations:** 1 Key Laboratory of Anti-inflammatory and Immune Medicine, Ministry of Education; Collaborative Innovation Center of Anti-inflammatory and Immune Medicine, Institute of Clinical Pharmacology, Anhui Medical University, Hefei, China; 2 Department of Pharmacology, University of California, Davis, Davis, California, USA; 3 Department of Veterans Affairs Northern California Healthcare System, Mather, California, USA; 4 School of Pharmaceutical Sciences and the Fifth Affiliated Hospital, Guangzhou Medical University, Guangzhou, China; 5 Department of Cardiology, Sun Yat-sen Memorial Hospital, Sun Yat-sen University, Guangzhou, China; 6 Department of Emergency Medicine, The First Affiliated Hospital of USTC, Division of Life Sciences and Medicine, University of Science and Technology of China, Hefei, China

**Keywords:** diabetic cardiomyopathy, excitation contraction coupling, myocytes, PDE4D, SERCA2a

## Abstract

**Background and Purpose:**

Sarcoplasmic reticulum Ca^2+^-ATPase (SERCA2a) is impaired in heart failure. Phosphodiesterases (PDEs) are implicated in the modulation of local cAMP signals and protein kinase A (PKA) activity essential for cardiac function. We characterise PDE isoforms that underlie decreased activities of SERCA2a and reduced cardiac contractile function in diabetic cardiomyopathy.

**Experimental Approach:**

Wild type mice were fed with either normal chow or a highfat diet (HFD). Cardiomyocytes were isolated for excitation–contraction coupling (ECC), fluorescence resonant energy transfer PKA biosensor and proximity ligation assays.

**Key Results:**

The upregulated PDE4D3 and PDE4D9 isoforms in HFD cardiomyocytes specifically bound to SERCA2a but not ryanodine receptor 2 (RyR2) on the sarcoplasmic reticulum (SR). The increased association of PDE4D isoforms with SERCA2a in HFD cardiomyocytes led to reduced local PKA activities and phosphorylation of phospholamban (PLB) but minimally effected the PKA activities and phosphorylation of RyR2. These changes correlate with slower calcium decay tau in the SR and attenuation of ECC in HFD cardiomyocytes. Selective inhibition of PDE4D3 or PDE4D9 restored PKA activities and phosphorylation of PLB at the SERCA2a complex, recovered calcium decay tau, and increased ECC in HFD cardiomyocytes. Therapies with PDE4 inhibitor roflumilast, PDE4D inhibitor BPN14770 or genetical deletion of PDE4D restored PKA phosphorylation of PLB and cardiac contractile function.

**Conclusion and Implications:**

The current study identifies upregulation of specific PDE4D isoforms that selectively inhibit SERCA2a function in HFD-induced cardiomyopathy, indicating that this remodelling can be targeted to restore cardiac contractility in diabetic cardiomyopathy.

## 1 | INTRODUCTION

Diabetic cardiomyopathy (DCM), cerebrovascular disease and renal impairment are major lethal outcomes of diabetes. Hamby first proposed the concept of DCM, and it is mainly characterised by left ventricular diastolic dysfunction and cardiac insufficiency independent of coronary heart disease, hypertension or heart valve disease (Hamby et al., 1974). The pathogenesis of DCM remains unclear, and it is generally believed that metabolic disorders, impaired mitochondrial function and an imbalance of calcium regulation in cardiomyocytes are involved (Jia et al., 2018). Currently, only symptomatic-based treatments are implemented to treat DCM, consisting primarily of blood glucose, lipid and pressure control; insulin resistance improvement; and cardiac remodelling prevention with β-blockers or angiotensin-converting enzyme inhibitors (Borghetti et al., 2018). However, the mortality is greatly increased once diabetic patients develop heart failure (Arnold et al., 2016). Therefore, understanding the pathogenesis of DCM and exploring new therapeutic targets is of great significance.

The efficient cycling of calcium between the cytoplasm and the sarcoplasmic reticulum (SR) is essential for the contraction and relaxation of cardiomyocytes. SR SERCA2a and ryanodine receptor 2 (RyR2) coordinate this physiological process; both are precisely regulated by cyclic adenosine monophosphate (cAMP)- protein kinase A (PKA) signalling induced by β-adrenoceptors (Zhang et al., 2013). Phosphodiesterases (PDEs) are critical regulators of cAMP signals and PKA activities in hearts. Studies have demonstrated that each isoform of PDE controls a specific pool of cAMP at a distinct subcellular localisation, resulting in differential cAMP–PKA signalling at functional micro/nanodomains in cardiomyocytes (Folkmanaite & Zaccolo, 2023; Lin et al., 2023; Tomek & Zaccolo, 2023). Of note, a specific PDE4D3 isoform associates with RyR2 (Lehnart et al., 2005), while PDE3A is linked to SERCA2a in healthy cardiac contractility (Ahmad et al., 2015; Beca et al., 2013). Previous evidence shows that changes in compartmentalised cAMP–PKA signalling at RyR2 and myofilaments exert important pathological effects in heart failure induced by stenosis and aortic insufficiency (Barbagallo et al., 2016; Berisha et al., 2021). However, little is known about how PDEs are involved in the pathological remodelling of cAMP signals and PKA activities on SERCA2a nanodomains during heart failure. PDE may contribute to a loss of SERCA2a function, reduced calcium reuptake into the SR and impaired cardiac relaxation in DCM.

We have recently reported that insulin receptors and β_2_-adrenoceptors form a complex in hearts (Fu et al., 2015; Fu, Xu, et al., 2014). Hyperinsulinemia in high-fat diet (HFD) feeding mice activates cardiac insulin receptors and triggers insulin receptor substrate-dependent recruitment of G protein-coupled receptor kinase 2 (GRK2) to the β_2_-adrenoceptor (Q. Wang et al., 2017). The phosphorylated β_2_-adrenoceptor subsequently promotes PDE4D expression via an extracellular signal-related kinase (ERK) signalling pathway, reducing cAMP levels and PKA activities in cardiomyocytes (Q. Wang et al., 2017). Eleven isoforms of PDE4D have been detected, including cardiac-expressed PDE4D3, PDE4D5, PDE4D8 and PDE4D9. All of these belong to the long PDE4D isoforms, each with a unique N-terminus followed by upstream conserved region 1 (UCR1) and upstream conserved region 2 (UCR2) domains. The unique N-terminus of each isoform facilitates anchoring to other specific molecules and produces an intracellularly precise location (Derici et al., 2019). We hypothesise that the upregulation of specific PDE4D isoforms in DCM hearts leads to reduced phosphorylation of phospholamban (PLB) and impaired SERCA2a function, contributing to cardiomyocyte E–C coupling (ECC) dysfunction.

In the present work, we identified the specific cardiac PDE4D isoforms upregulated in HFD cardiomyocytes. We then implemented fluorescence resonance energy transfer (FRET) biosensors to characterise the impacts of PDE4D isoforms on PKA activities at distinct subcellular nanodomains in cardiomyocytes. The results establish that the upregulated PDE4D isoforms selectively impair local PKA activities at the SERCA2a nanodomains. The effects of PDE4D isoforms were further probed with isoform-specific dominant negative peptides by measuring downstream local PKA activities, protein phosphorylation, calcium handling and myocyte contractility. This work raises potential DCM treatment strategies that inhibit PDE4D isoforms with PDE4D-specific inhibitors or directly target the pathogenic PDE4D3 and PDE4D9 isoforms associated with SERCA2a in diabetic hearts.

## 2 | METHODS

### 2.1 | Reagents and materials

All reagents and materials were obtained from Millipore–Sigma (St. Louis, MO, USA) unless otherwise specified.

### 2.2 | Animals

All animal studies and experimental protocols (20956 and 20957) were approved by the Institutional Animal Care and Use Committees (IACUC) of the University of California at Davis and complied with the guidelines of National Institutes of Health and ARRIVE guidelines. Eighty 6–8 week old male wild type (WT) C57BL/6J mice were purchased from Jackson Laboratories (Sacramento, CA, USA). PDE4D knockout (PDE4D^−/−^) mice were purchased from GemPharmatech Co., Ltd (Nanjing, Jiangsu, China, Strain NO. T032409). All mice were maintained in a standard room with controlled temperature, humidity and 12-h light–dark cycle. Animal studies are reported in compliance with the ARRIVE guidelines (Percie du Sert et al., 2020) and with the recommendations made by the *British Journal of Pharmacology* (Lilley et al., 2020).

### 2.3 | Peptides

The myristoylated NT peptides of PDE4D3 (MMHVNNFPFR RHSWICF), PDE4D5 (MAQQTSPDTLTVPEVDNPHCPNPWLNEDLVK SLRENLLQHEKSKTARKSVSPKLSPVISPRNSPRLLRRMLLSSNIPKQRR FTVAHTCF), PDE4D8 (MAFVWDPLGATVPGPSTRAKSRLRFSKSYSF) and PDE4D9 (MSIIMKPRSRSTSSLRTAEAVCF) were synthesised by ChinaPeptides (Shanghai, China). Chemicals are from Sigma unless specified.

### 2.4 | DCM model induction and treatment

Mouse DCM model was induced by HFD feeding. Eight-week old C57BL/6J WT and PDE4D^−/−^ mice were randomly split into normal chow (NC) and HFD feeding groups. The HFD group was given a 60% fat diet (D12492, Research Diets Inc., New Brunswick, NJ, USA). The NC group was fed a matching low-fat rodent diet (D12450J, Research Diets Inc.) with 3.8 kcal g^−1^ containing 20% of calories from protein, 70% of calories from carbohydrate and 10% of calories from fat. The mice were given ad libitum access to food and water for 4.5 months (Q. Wang et al., 2017). The treatment was initiated when HFD mice developed left ventricular diastolic dysfunction (Q. Wang et al., 2017). The HFD group was randomly divided into a vehicle (saline or 0.05% sodium carboxymethylcellulose) group, a 10 mg kg^−1^ PDE4 inhibitor roflumilast treatment group (dissolved in saline; Elmasry et al., 2022; Hatzelmann et al., 2010), or a 0.03 mg kg^−1^ PDE4D inhibitor BPN14770 (Selleck, Houston, TX, USA) treatment group (dissolved in 0.05% sodium carboxymethylcellulose; Y. Wang et al., 2020). The mice were treated by daily gavage for 4 weeks. PDE4D^−/−^ and WT littermates were fed with either NC or HFD for 4.5 months.

### 2.5 | Cardiomyocyte isolation and in vitro treatment

Adult ventricular myocytes were isolated using the method as previously described (Reddy et al., 2018). Anaesthesia of mice was induced by 2% isoflurane. The heart was quickly removed, and the aortic cannulation was performed under an operating microscope. After perfusing with the cannulation buffer for 4 min at a controlled speed of 3 ml min^−1^, the heart was predigested with a combined solution of 15 ml dilute collagenase solution and 10 ml cannulation buffer. Then the heart was fully digested by recirculating 20 ml full collagenase solution until the heart became soft. The ventricular portion of the heart was cut off and gently torn into small pieces using forceps in a dish and then transferred into a 15-ml tube with 5 ml of full collagenase solution. The supernatant was transferred into a 5 ml stopping buffer in a new tube and was centrifuged at 1000 g for 1 min. The myocytes were resuspended in the cannulation buffer with gradient concentration of calcium up to 1 mM. Myocytes were counted and 40,000 cells per ml in Mouse FRET media with 10% FBS were seeded in dishes coated with mouse purified Laminin (Life Technologies, Grand Island, NY, USA), as previously described (Reddy et al., 2018). The media was changed 4 h later to remove the unattached cells.

The formulas of the solutions are listed below. Cannulation buffer: NaCl 120 mM, NaH_2_PO_4_ 1.2 mM, KCl 5.4 mM, MgSO_4_ 1.2 mM, NaHCO_3_ 20 mM, glucose 5.6 mM, taurine 20 mM, 2,3-butanedione monoxime 10 mM and PH 7.33. The buffer was bubbled with 95% oxygen and 5% carbon dioxide for 10 min before use. Dilute collagenase solution: 2.5 mg type II collagenase (Worthington Biochemical, Lakewood, NJ, USA), 0.5 mg type XIV protease and 0.1% bovine serum albumen (BSA). Full collagenase solution: 10 mg type II collagenase, 2 mg type XIV protease, 50 μM CaCl_2_ and 0.1% BSA. Stopping buffer: 12.5 μM CaCl_2_ and 0.5 ml fetal bovine serum (FBS). M1018 media: 10.7gM1018 Minimum Essential Medium Eagle, 0.35 g NaCHO_3_, 1% penicillin–streptomycin, PH 7.4. Mouse FRET Media: 1 × M1018, 1 × PSG, 0.2% BSA, 4 mM NaHCO_3_, 10 mM HEPES and 6.25 μM blebbistatin. All the buffers were filtered through a 0.22-μm filter.

### 2.6 | Cardiomyocyte contractility and calcium transient determination

The sarcomere contractile function and calcium transient amplitude and tau were determined as previously reported (Q. Wang et al., 2017; West et al., 2019). Freshly isolated ventricular myocytes were stained with Fluo-4AM (5 μM, Thermo Fisher Scientific, Waltham, MA) in the dark for 15 min at room temperature (RT), and then rinsed with PBS without calcium. The cells were gently added into 3 ml of beating buffer in a dish placed on the stage of the Zeiss fluorescence inverted microscope system (AX10, Dublin, CA, USA). Myocytes were paced using an SD9 stimulator (Grass Technology, Warwick, RI, USA) with 1 Hz at 50 V. Metamorph software was used to image contractility (exposure time 25 ms and total 200 frames) for sarcomere shortening (SS) calculation. Fluo-4AM imaging was applied for calcium transient amplitude and tau analysis with GaiLab software (Q. Wang et al., 2017; West et al., 2019).

### 2.7 | FRET assay

The FRET assay was carried out following the method reported before (Barbagallo et al., 2016). After initial 4-h plating of myocytes on laminin-coated coverslips, the media was changed to serum free mouse FRET media (PH 7.35, filtered, Sigma) supplemented with individual biosensors for 36 h before recording on a Leica inverted fluorescence microscope (DMI3000 B, Buffalo Grove, IL, USA). Myocytes infected with adenovirus expressing Reg-AKAR3 (detecting cytoplasmic PKA; S. Liu et al., 2012), PM-AKAR3, (detecting PM PKA; S. Liu et al., 2012), FKBP-AKAR3 (detecting RyR2 PKA; Xu, Wang, et al., 2022), SR-AKAR3 (detecting SRRCA2a PKA; S. Liu et al., 2012) or TNT-AKAR3 (detecting myofilament PKA; Barbagallo et al., 2016) to detect whole cell and subcellular PKA activities. Myocytes were transferred to a dish containing PBS without calcium and recorded every 30 s with a 40X objective lens using Metafluor software (Molecular Devices, Sunnyvale, CA, USA). Cyan fluorescent protein (CFP) images were obtained by filter 475DF40; and yellow fluorescent protein (YFP) was imaged by filter 535DF25. The background was subtracted before calculating the intensity ratio of CFP/YFP signals. An increase in the ratio indicates the activation of PKA.

### 2.8 | Co-immunoprecipitation

NC and HFD heart tissues were homogenised by 1 ml of lysis buffer (25 mM Hepes, 1% Triton X-100, 150 mM NaCl, 5 mM EDTA, 10% glycerol) and centrifuged at 16000 g for 30 min at 4°C. The supernatant (900 ul) was incubated 2 μg of anti-SERCA2 at 4°C overnight. After a brief spin to clear up the aggregates, 40 ul of protein G beads were added and mixed with supernatant for 4 h. The beads were harvested and rinsed with lysis buffer for five times. The bead-bound proteins were solubilised with 40 μl 2× SDS sample loading buffer (#161–0747, Bio-Rad Laboratories, Hercules, CA, USA) and subjected to western blots to probe SERCA2a and PDE4 proteins.

### 2.9 | Western blotting

Myocytes from NC- and HFD-fed mice were treated with 1 μM of rolipram (Roli, Sigma, MO, USA) for 10 min to detect the effects of Roli on PKA phosphorylation of PLB and RyR2. To detect the effects of Roli on isoproterenol (ISO, Sigma)-induced phosphorylation of PLB and RyR2, myocytes were treated with Roli (100 nM) for 10 min prior to 10 min ISO (100 nM) stimulation. Myocytes were also treated with 10 μM of a given PDE4D isoform NT peptide together with 100 nM ISO for 10 min as indicated. Myocytes and heart tissues from feeding mice were lysed with Radio Immunoprecipitation Assay (RIPA) buffer supplemented with proteinase and phosphatase inhibitors. Immunoblotting was applied to detect the expression of PLB (Thermo Fisher Scientific Cat# MA3–922, RRID:AB_2252716), pPLB at Ser16 (Badrilla Cat# A010–12AP, RRID:AB_3665194) pPLB at Thr17 (Badrilla Cat# A010–13AP, RRID:AB_3665193), SERCA2a (Abcam Cat# ab2861, RRID:AB_2061425), TnI (Cell Signalling Technology Cat# 4002, RRID: AB_2206278), pTnI at Ser23/24 (Cell Signalling Technology Cat# 4004, RRID:AB_2206275), pRyR2 at Ser2808 (Abcam Cat# ab59225, RRID:AB_946327), pRyR2 at Ser2814 (Badrilla Cat# A010–31AP, RRID:AB_3665192), RyR2 (Santa Cruz Biotechnology Cat# sc376,507, RRID:AB_11149759), PDE2A (Santa Cruz Biotechnology Cat# sc-25565, RRID:AB_2268159), PDE3A (PDE3A-CT, 1098–1,115, NIH), PDE4A (Gift from Prof. Marco Conti), PDE4B (Gift from Prof. Marco conti), PDE4D (Abcam Cat# ab14614, RRID:AB_2161095), PDE5A (Cell Signalling Technology Cat# 2395, RRID:AB_2161269), PDE4D3 (Fabgennix Cat# PD4D3–431AP, RRID:AB_3665191), PDE4D5 (Fabgennix Cat# PDE4D5 Antibody, RRID:AB_3665190), PDE4D8 (Fabgennix Cat# PD4D8–481AP, RRID:AB_3665189), PDE4D9 (Customised by Abmart, China), GAPDH (Proteintech Cat# 60004–1-Ig, RRID:AB_2107436) and γ-tubulin (Sigma-Aldrich Cat# T6557, RRID:AB_477584). DyLight 680 (Rockland Cat# 18–4417-32, RRID:AB_2610840) and 800 (Rockland Cat# 18–4516-32, RRID:AB_2610841) secondary antibodies were used for multi-colour detection. Polyvinylidene fluoride (PVDF) membranes were scanned on Odyssey imaging systems (LI-COR Biosciences Lincoln, NE, USA), and the western blots were semi-quantified with ImageJ. The Immuno-related procedures used comply with the recommendations made by the *British Journal of Pharmacology* (Alexander et al., 2018).

### 2.10 | Proximity ligation assay (PLA)

PLA was performed according to instructions of the Duolink^®^ In Situ PLA Kit (Cat. No. DUO92007, Sigma-Aldrich). Mouse monoclonal SERCA2a and RyR2 antibodies, and rabbit anti-PDE4D3, PDE4D5, and PDE4D9 antibodies (De Arcangelis et al., 2009; Fu, Kim, et al., 2014) were used to detect the in situ interaction between indicated PDE4D isoform and SERCA2a or RyR2 in adult myocytes from NC- and HFD-fed mice. The negative control was run by probing only one antibody. Briefly, myocytes were fixed and permeabilised, followed by blocking in Duolink blocking reagent at 37°C in a humidity chamber for 1 h. After probing with two primary antibodies, the cells were incubated with PLUS and MINUS PLA probes at 37°C for 1 h. Thereafter, myocytes were incubated with ligation buffer for 30 min and amplification solution for 100 min, and then were mounted using Duolink In Situ Mounting Medium with DAPI. The stainings were imaged with a Zeiss confocal microscope (LSM 700, Gottingen, Germany), and the number of the interactions was quantified with ImageJ (NIH).

### 2.11 | Echocardiography

Echocardiography was performed using a Vevo 2100 imaging system (Toronto, ON, Canada) with a 22–55 MHz MS550D transducer. Mice were anaesthetised by 2% isoflurane induction, and 1% isoflurane was used to maintain anaesthesia. The body temperature, respiratory rate and electrocardiogram of the mice were assessed and monitored during imaging. The heart rate of the mice was maintained at 400–500 beats per minute. Systolic function parameters including ejection fraction (EF) and fractional shortening (FS) were measured in the M-mode of the parasternal short-axis imaging plane near the papillary muscles. Tissue Doppler imaging modality was applied to measure diastolic function, and the main parameter obtained was peak tissue Doppler of myocardial relaxation velocity at the mitral valve annulus during early diastole (E’), peak tissue Doppler of myocardial relaxation velocity at the mitral valve annulus during late diastole (A′) and isovolumic relaxation time (IVRT).

### 2.12 | Intraperitoneal glucose and insulin tolerance test

Mice were separated to 1 mouse per cage at 8 AM and fasted for 6 h in a quiet experimental area to minimise the stress. After fasting, mice were injected intraperitoneally with a sterile glucose solution (1 mg g^−1^) or a sterile insulin solution (0.75 U kg^−1^). A sharp blade was used to incise the tail tip, and 0.5 μl blood sample was taken from the tail vein gently by massaging at 0, 15, 30, 60 and 120 min post-injection and measured using a standard glucometer (Bayer, Pittsburgh, PA). The mice were resting in the cages with accessible to water between the time points.

### 2.13 | Tissue collection and histology

Mice were euthanised after anaesthesia with isoflurane, and hearts were flushed with 1 M KCl, perfused with 4% paraformaldehyde and saline, and fixed in 4% paraformaldehyde for 24 h. The hearts were then embedded in paraffin and cut into 5 μm sections. Tissue sections were stained with Masson’s Trichrome (Sigma-Aldrich), according to the manufacturer’s instructions. The ratio of blue areas representing collagen to the overall myocardial tissue in the images was analysed using ImageJ software to quantify the extent of myocardial fibrosis. Cardiac tissue sections were also stained using iFluor^™^ 488-Wheat Germ Agglutinin (WGA, AAT Bioquest, Sunnyvale, CA, USA) according to the manufacturer’s protocol. In brief, after dewaxing and dehydrating the heart paraffin sections with gradient alcohol, 100 μl of wheat germ agglutinin (WGA) conjugate working solution was added to the tissue sections and incubated for 30 min at room temperature and protected from light. Images were taken on an SP8 lightning confocal microscope (Leica, Wetzlar, Germany). The average cross-sectional area of 30 cardiomyocytes per heart was calculated and used as a data point. Liver and perirenal fat were collected and weighed.

### 2.14 | Statistical analysis

Data and statistical analysis complied with the recommendations of the *British Journal of Pharmacology* on experimental design and analysis in pharmacology (Curtis et al., 2022). Data are expressed as mean ± standard error of the mean, variance analyses were performed with SPSS 13.0 (IBM, Armonk, NY, USA), and the statistical analysis was conducted using GraphPad Prism 6 software (La Jolla, CA, USA). The comparisons between two groups or multiple groups were determined by two-tailed unpaired *t*-test and one-way ANOVA with Tukey posttest, respectively. Post-hoc tests were run only if F achieved *P*<0.05 and there was no significant variance inhomogeneity. *P*-values < 0.05 were considered significantly different. Sample size (*n*) represents the number of animals used for in vivo studies, as well as for cardiomyocytes isolation.

### 2.15 | Nomenclature of targets and ligands

Key protein targets and ligands in this article are hyperlinked to corresponding entries in the IUPHAR/BPS Guide to PHARMACOLOGY http://www.guidetopharmacology.org and are permanently archived in the Concise Guide to PHARMACOLOGY 2023/2024 (Alexander, Christopoulos et al., 2023; Alexander, Fabbro et al., 2023; Alexander, Mathie et al., 2023).

## 3 | RESULTS

### 3.1 | Upregulated PDE4D impairs adrenergic signalling at the SERCA2a nanodomains, calcium recycling and contractile function in diabetic cardiomyocytes

**FIGURE 1:**
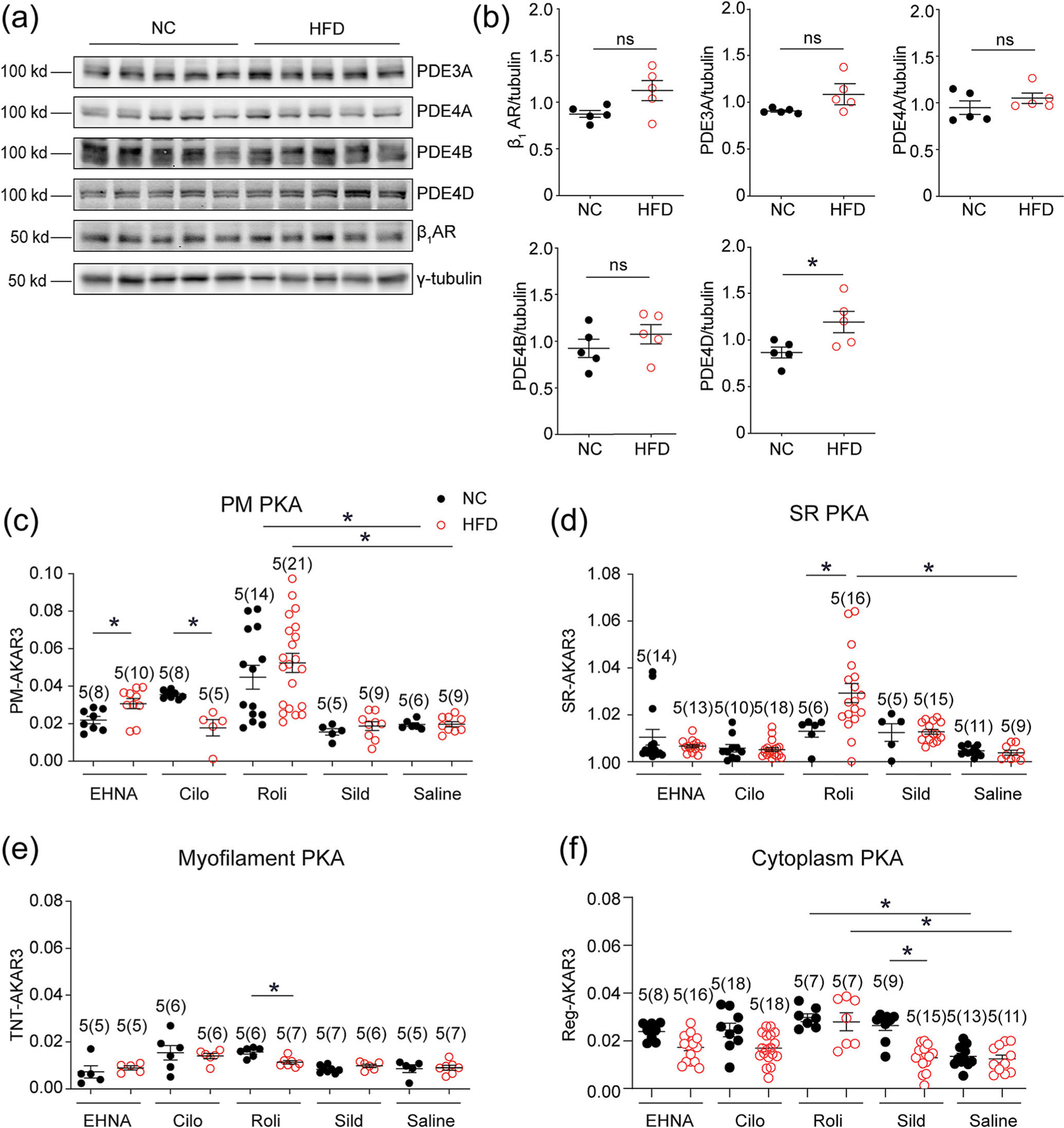
High-fat diet (HFD) feeding induces changes in PDE expression associated with subcellular nanodomains in cardiomyocytes. (a and b) WT mice were fed with a HFD or normal chow (NC) diet for 4.5 months. The heart tissues were harvested for western blot analysis to detect the expression of β1-adrenoceptor, PDE3A, 4A, 4B, 4D and γ-tubulin. The quantification of western blots is presented in dot plots. (c–f) Myocytes were isolated and infected with adenoviruses expressing different AKAR3 biosensors (PM, SR, TNT, Reg). Myocytes were treated with EHNA (10 mM), cilostamide (Cilo) (1 mM), rolipram (Roli) (10 mM) and sildenafil (Sild) (1 mM) as indicated. The changes in FRET ratio were recorded, and the maximal changes in FRET ratio were plotted. * P < 0.05 by Student *t* test in Figure 1b and by two-way ANOVA followed by Tukey’s test in other panels.

β_1_-Adrenoceptor signalling was impaired, manifesting as reduced ECC and contractility in myocytes from DCM induced by HFD feeding relative to those from mice fed with NC (Q. Wang et al., 2017). However, cardiac β_1_-adrenoceptor expression was found to be preserved in mice with HFD feeding for 4.5 months even though both diastolic and systolic dysfunction were displayed (Figure 1a and b, Table S1). At this time point, HFD feeding increased body weight, upregulated serum insulin levels and impaired glucose metabolism and insulin tolerance (Figure S1a–d). We have previously shown that HFD promotes PDE4D expression through the hyperinsulinemia-mediated insulin receptor-β_2_-adrenoceptor-GRK2-ERK pathway (Q. Wang et al., 2017). The elevated PDE4D compromises adrenergic stimulation of PKA activity in adult left ventricular myocytes (AVMs) and leads to impaired ECC in HFD-fed mice. While cardiac PDE4D was elevated in hearts after HFD feeding, PDE4A, PDE4B, PDE3A and PDE2A displayed an equivalent expression in NC and HFD hearts (Figure 1a and b) (Q. Wang et al., 2017). Recent studies report that a pool of intracellular β_1_-adrenoceptors located at the SR is essential for PKA-mediated phosphorylation of PLB and contributes to the contractility of myocytes in response to the catecholamine stimulation (Y. Wang et al., 2021). We hypothesised that the elevated expression of PDE4D may selectively target the PLB/SER-CA2a complex in HFD hearts. Directional distribution of subcellular FRET-based PKA activity biosensors (Figure S2a,b) were applied as proxy readouts to examine the subcellular distribution of upregulated PDE4D activities when a selective PDE4 inhibitor Roli was applied. Selective inhibitors against other cardiac PDE enzymes, including PDE2 (erythro-9-(2-Hydroxy-3-nonyl) adenine, EHNA), PDE3 (cilostamide, Cilo) and PDE5 (sildenafil, Sild), were examined as comparisons. PDE4 activities were primarily associated with the plasma membrane (PM) in NC and HFD myocytes (Figure 1c). Notably, PDE4 activities were markedly elevated at the SR SERCA2a complex in HFD myocytes compared with NC controls, while the effects of PDE2, PDE3 and PDE5 inhibitors on the PKA activities at the SERCA2a were similar between HFD and NC AVMs (Figure 1d). When treated with Roli, the PKA activities were slightly reduced at the myofilaments and did not change in the bulky cytoplasm in HFD myocytes compared with NC controls (Figure 1e and f). Additionally, the PKA activities at the PM were enhanced in HFD myocytes compared with NC controls when treated with EHNA but were decreased when treated with Cilo. The cytoplasm PKA activities induced by Sild were reduced in HFD myocytes compared with NC controls (Figure 1c and f). These FRET data support that the upregulated PDE4D isoforms are selectively distributed at the SR in HFD myocytes.

We examined the phosphorylation and expression levels of proteins localised at the distinct subcellular compartments in HFD hearts. The phosphorylation of PLB at Ser 16, which is a cAMP–PKA-dependent site (S. Liu et al., 2012), was markedly reduced in HFD-fed mice (Figure S3a,b), together with a reduced expression of SERCA2a (Figure S3A–D). However, the phosphorylation of RyR2 at Ser 2808 by PKA (Fischer et al., 2013; Houser, 2014) was not significantly altered in HFD hearts when compared to NC controls (Figure S3a,e), despite the fact that PLB and RyR2 are localised at the same subcellular organelle. However, the Ca^2+^/CaM-dependent protein kinase II (CaMKII)-dependent phosphorylation of PLB at Thr 17 and RyR2 at Ser 2814 was increased in HFD hearts (Figure S3a,c,f), consistent with elevated CaMKII activities in cardiac diseases (M. E. Anderson et al., 2011). The phosphorylation of troponin I (TnI) at Ser 23/24 was decreased in the HFD-fed hearts compared with NC controls (Figure S3A,G). These data indicate that upregulated PDE4D may be located at the SERCA2a to selectively desensitise adrenergic-PKA signalling in diabetic cardiomyocytes and contribute to the reduced activities of the calcium pump.

**FIGURE 2:**
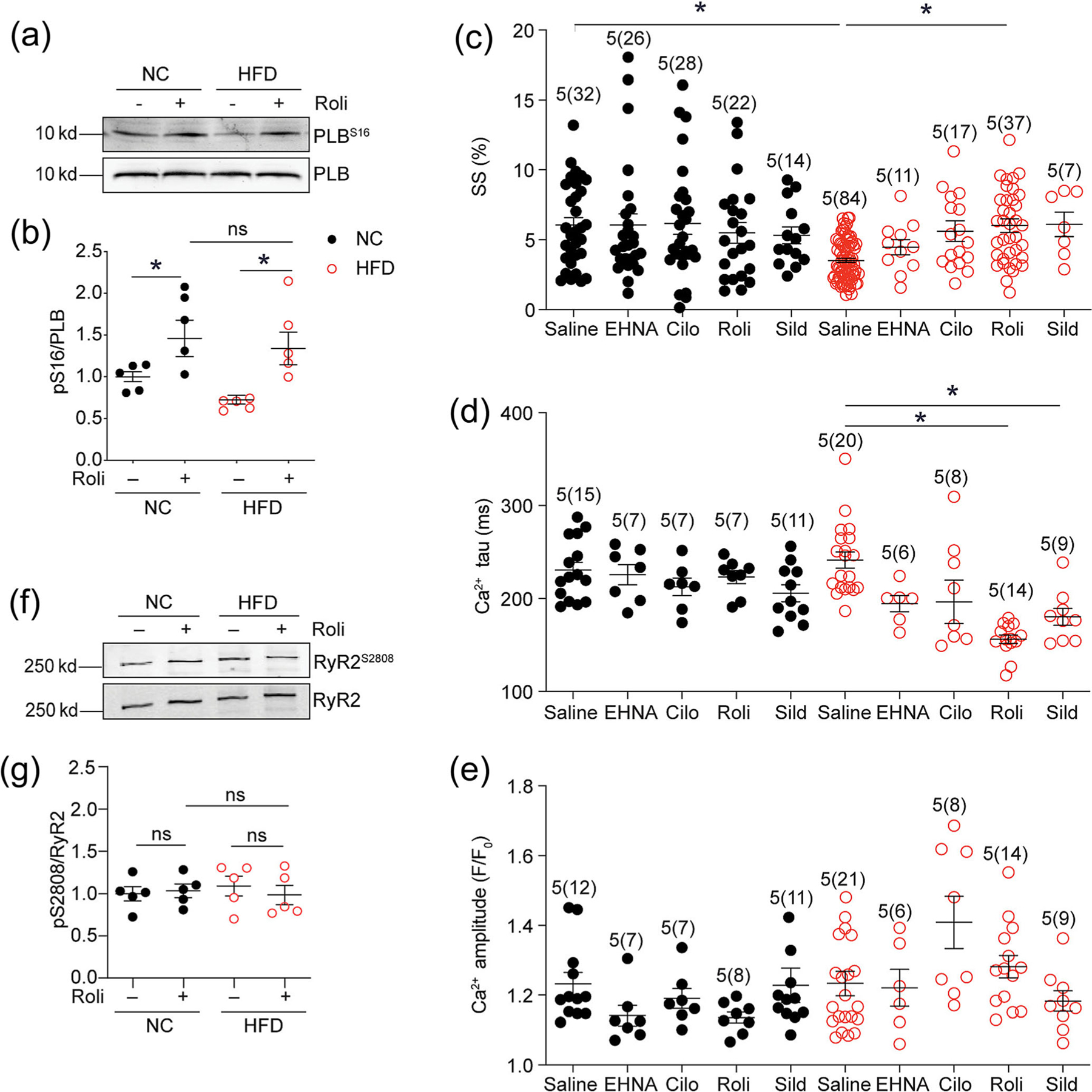
Inhibition of PDE4 rescues PKA phosphorylation of phospholamban and SR calcium load in high-fat diet (HFD)-fed cardiomyocytes. (a, b, f, g) Cardiac myocytes isolated from normal chow (NC) or HFD mice were treated with Roli (10 μM) for 10 min. Cells were lysed for western blots to detect the levels of phosphorylation of PLB at Ser16 and RYR2 at Ser2808. The quantification of western blot is shown in dot plots. (c–e) Cardiac myocytes loaded with calcium dye Fluo-4AM and paced at 1 Hz. Myocytes were treated with EHNA (10 μM), cilostamide (Cilo) (1 μM), rolipram (Roli) (10 μM) and sildenafil (Sild) (1 μM) as indicated. Calcium signals and SS were recorded. The maximal changes in sarcomere shortening (SS), calcium decay tau and transient amplitude were plotted. *P < 0.05 by two-way ANOVA followed by Tukey’s test.

To further validate that the distribution of PDE4D at the SER-CA2a complexes, myocytes isolated from NC- and HFD-fed mice were treated with the PDE4 inhibitor Roli. Treatment with Roli increased the phosphorylation of PLB at S16 in HFD myocytes (Figure 2a and b). Accordingly, Roli significantly accelerated calcium decay tau associated with normalised contractile shortening in HFD myocytes (Figures 2c–e and S4 and S5). In comparison, Roli treatment did not affect the phosphorylation of RyR2 at the PKA site of S2808 (Figure 2f and g). These data indicate a highly selective targeting of upregulated PDE4D isoforms at SERCA2a complexes, which disrupts calcium handling in HFD myocytes.

### 3.2 | Inhibition of PDE4 restores the β_1_-adrenoceptor-induced PKA activities at the SERCA2a nanodomains and improves calcium recycling and contractile function in HFD-fed myocytes

**FIGURE 3:**
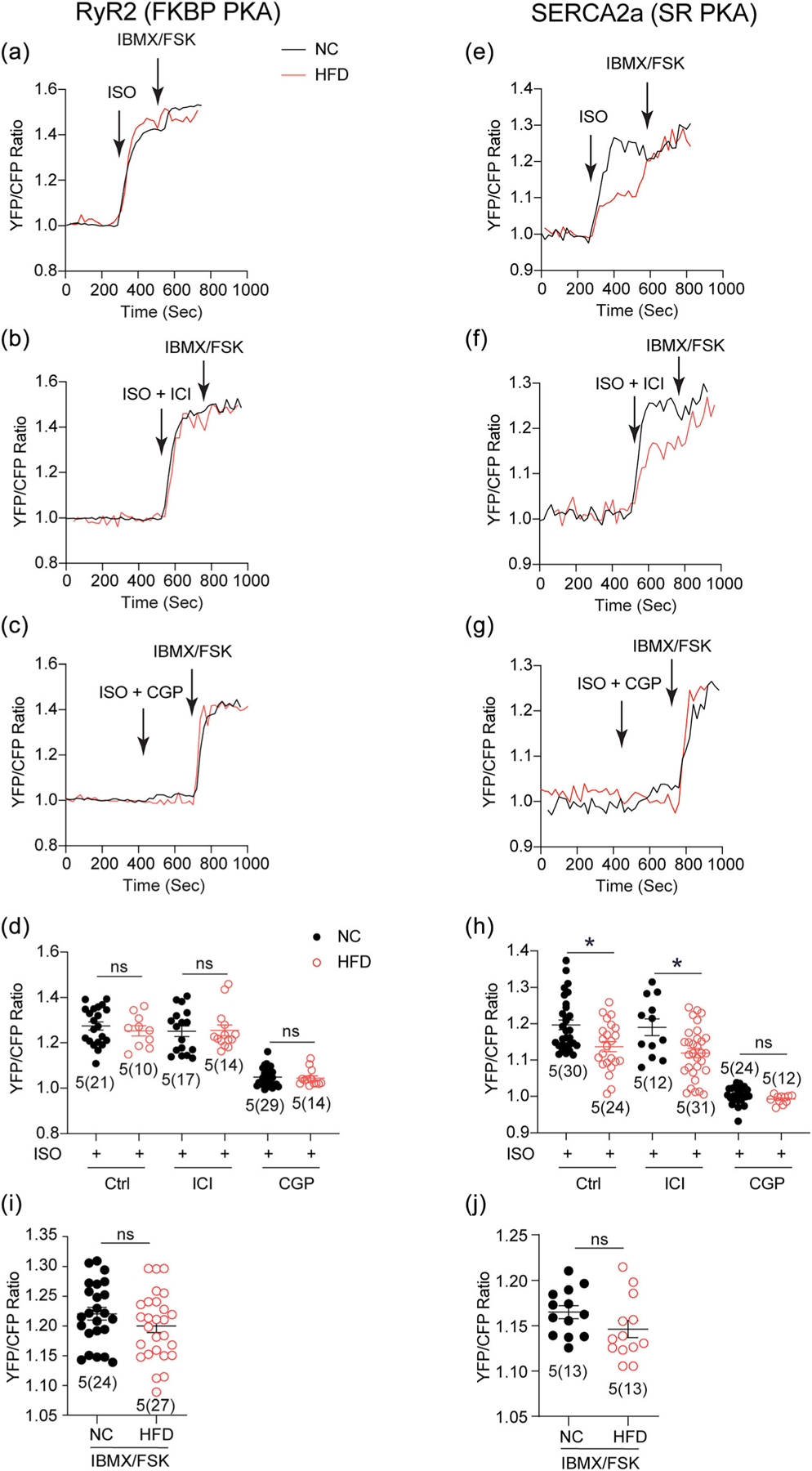
The β_1_-adrenoceptor-induced PKA activity of the SERCA2a nanodomains is reduced in high-fat diet (HFD)-fed cardiomyocytes. (a–j) Isolated myocytes from normal chow (NC) and HFD fed mice were infected with adenoviruses expressing RyR2 and SERCA2a associated FKBP and SR AKAR3 biosensors. Myocytes were pretreated with CGP20712a (300 nM) or ICI11811 (100 nM) before stimulation with isoproterenol (ISO, 100 nM) and followed by addition of forskolin (FSK) (10 μM) and IBMX (100 μM). The time courses and the maximal changes in FRET ratio over the baseline were calculated and plotted. *P < 0.05 by two-way ANOVA followed by Tukey’s test. Ctrl, control.

To further analyse the adrenergic induced-PKA signalling dynamics within RyR2 and SERCA2a local SR nanodomains in diabetic cardiomyocytes, we applied the FKBP-AKAR3 biosensor targeted to RyR2 and SR-AKAR3 anchored to SERCA2a complexes (S. Liu et al., 2012; Liu et al., 2010; Xu, Wang, et al., 2022). In HFD AVMs, ISO induced similar increases in PKA activities at the RyR2 domains relative to those of NC controls (Figure 3a–d). However, HFD AVMs displayed significantly reduced ISO stimulation of PKA activities at the SERCA2a domains relative to NC controls (Figure 3e and h). The adrenergic-induced PKA activities were abolished by the β_1_-adrenoceptor blocker CGP20712a but not by the β_2_-adrenoceptor blocker ICI118551 in HFD myocytes (Figure 3f–h). As a control, the addition of the adenylyl cyclase stimulator forskolin (FSK) and general PDE inhibitor 3-isobutyl-1-methylxanthine (IBMX) had comparable PKA activities at RyR2 and SERCA2a local domains in NC and HFD AVMs, respectively (Figure 3i and j). Together, these data indicate that adrenergic signalling is selectively desensitised at the SERCA2a local domains in diabetic cardiomyocytes, and the β_1_-adrenoceptor rather than the β_2_-adrenoceptor is responsible for promoting PKA activities at the SERCA2a domains in control and diabetic cardiomyocytes.

**FIGURE 4:**
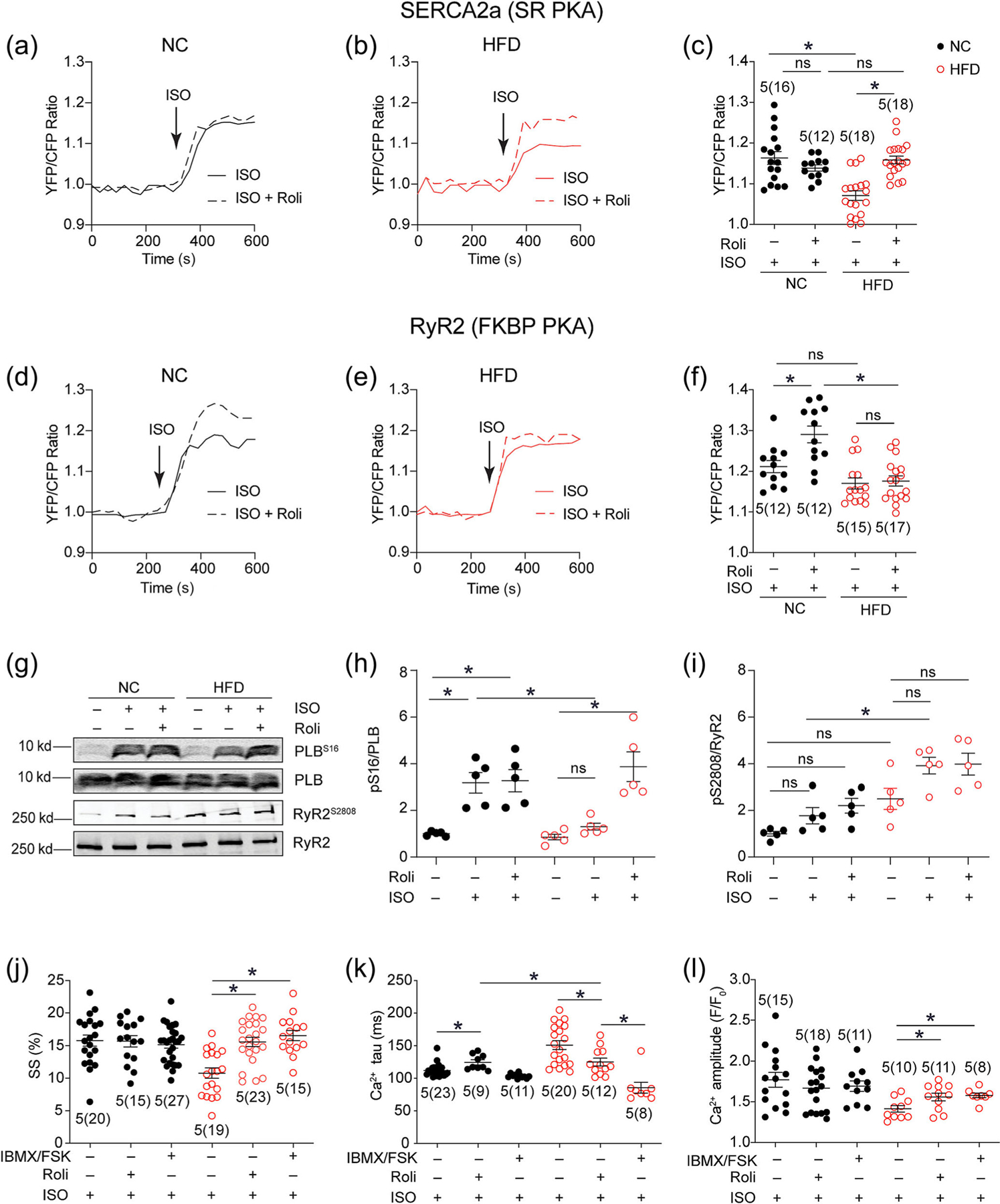
Inhibition of PDE4 restores β_1_-adrenoceptor-induced PKA activity at the SERCA2a nanodomains and improves calcium recycling and contractile function in high-fat diet (HFD)-fed myocytes. (a–f) Isolated myocytes from normal chow (NC) and HFD mice were infected with adenoviruses expressing RyR2 and SERCA2a associated FKBP and SR AKAR3 biosensors. After treatment with PDE4 inhibitor Roli (100 nM) or Veh, myocytes were stimulated with the β-adrenoceptor agonist ISO (100 nM). The time courses and maximal changes in FRET ratio was presented. (g–i) Isolated myocytes were stimulated with ISO (100 nM, 10 min) in the presence of rolipram (Roli) (100 nM) or Veh as indicated. Cell lysates were subjected to detection of phosphorylation of PLB at Ser16 and RyR2 at Ser2808. The western blots were quantified in dot plots. (j–l) Isolated myocytes were loaded with calcium dye Fluo-4 AM and paced at 1 Hz. Myocytes were stimulated with ISO (100 nM) in the presence and absence of Roli (100 nM) or Veh and followed by addition of IBMX (100 μM)/FSK (10 μM). Calcium signals and sarcomere shortening (SS) were recorded. Sarcomere shortening and calcium decay tau and transient amplitude were calculated and plotted. *P < 0.05 by two-way ANOVA followed by Tukey’s test.

The PDE4 inhibitor Roli effectively rescued the diminished β-adrenoceptor agonist ISO-induced PKA activities at SERCA2a domains in myocytes from HFD-fed mice (Figure 4a–c). There was little change in ISO-induced PKA activities at the RyR2 domains in HFD myocytes relative to NC controls; the addition of Roli did not influence ISO responses (Figure 4d–f). Consistent with the FRET data, the ISO-induced phosphorylation of PLB at the PKA site of S16 was significantly decreased in HFD myocytes, and Roli recovered ISO-induced phosphorylation of PLB (Figure 4g and h). In comparison, ISO induced higher levels of phosphorylation of RyR2 at the PKA site of S2808 in HFD myocytes relative to NC controls. Roli did not further enhance the levels of phosphorylation of S2808 (Figure 4g and i). Functionally, pretreatment with Roli effectively rescued ISO-induced contractility, calcium decay tau and transient amplitude of HFD myocytes (Figure 4j–l). Additional treatment with the adenylyl cyclase stimulator FSK and general PDE inhibitor IBMX promoted similar responses in PKA phosphorylation of PLB and RyR2, contractile shortening, and calcium transient amplitude in NC and HFD AVMs but with a faster calcium decay tau in HFD myocytes than NC controls (Figures S6 and 4j–l). These data show that the upregulated PDE4D isoforms differentially target SERCA2a but not RyR2 complexes to attenuate adrenergic responses.

### 3.3 | Inhibition of PDE4 improves the diastolic function of HFD-fed mice

**FIGURE 5:**
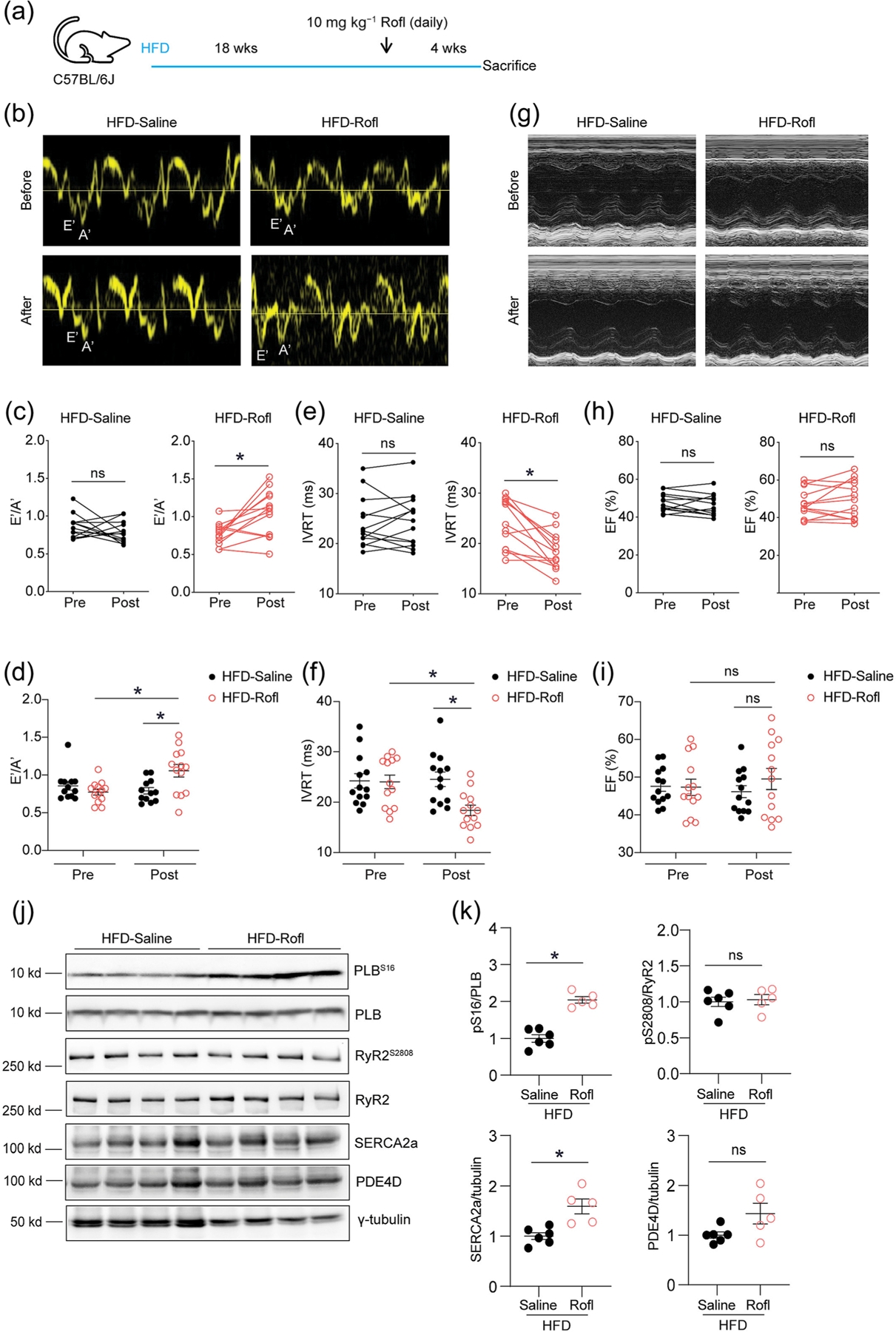
Inhibition of PDE4 improves diastolic function of high-fat diet (HFD)-fed mice. (a) Diagram shows that WT mice were fed wi7th HFD for 4.5 months and then treated with saline or roflumilast (Rofl) (10 mg kg^−1^) for 4 weeks. (b–i) Echocardiography was used to detect diastolic function parameters E′/A′ and isovolumic relaxation time (IVRT) and systolic function parameter ejection fraction (EF) in HFD mice after treatments. (j and k) After treatments, heart tissues from HFD mice were harvested for western blot analysis to detect the expression of PLB, SERCA2a, RyR2 and γ-tubulin, as well as phosphorylation of PLB at Ser16 and phosphorylation of RyR2 at Ser2808. The western blots were quantified and presented in dot plots. *P < 0.05 by one-way ANOVA followed by Tukey’s test in Figure 5d, f, i, and by Student *t* test in other panels.

We then attempted a therapy in the mouse model of HFD feeding with an FDA-approved PDE4 inhibitor, roflumilast (Rofl, 10 mg kg^−1^ day^−1^, 4 weeks) (Figure 5a). Mice underwent HFD feeding for 4.5 months to induce cardiac hypertrophy and diastolic dysfunction. Rofl therapy significantly improved diastolic parameters E′/A′ and IVRT in HFD mice over the saline controls (Figure 5b–f and Table S2) with minimally affected systolic parameter ejection fraction (Figure 5g–i and Table S2). Consistent with the functional improvement, therapy with Rofl significantly enhanced PKA phosphorylation of PLB and rescued the expression of SERCA2a but did not affect the phosphorylation of RyR2 at S2808 and the expression of PDE4D in HFD hearts (Figure 5j and k). Additionally, therapy with PDE4 inhibitor reduced the body weights but did not affect glucose tolerance in HFD mice (Figure S7A,C,D). Moreover, Rolf treatment did not affect the heart weights but reduced cardiac fibrosis in both interstitial and perivascular areas in HFD mice (Figure S7B,E,F). Additionally, Rolf treatment increased PDE3A expression but had no obvious effect on PDE2A, PDE4D and PDE5A expression in HFD hearts (Figure S7G,H). These data confirm that inhibition of PDE4 is a promising strategy for treating DCM.

### 3.4 | Upregulated PDE4D3 and PDE4D9 selectively associate with SERCA2a in HFD-fed myocytes

**FIGURE 6:**
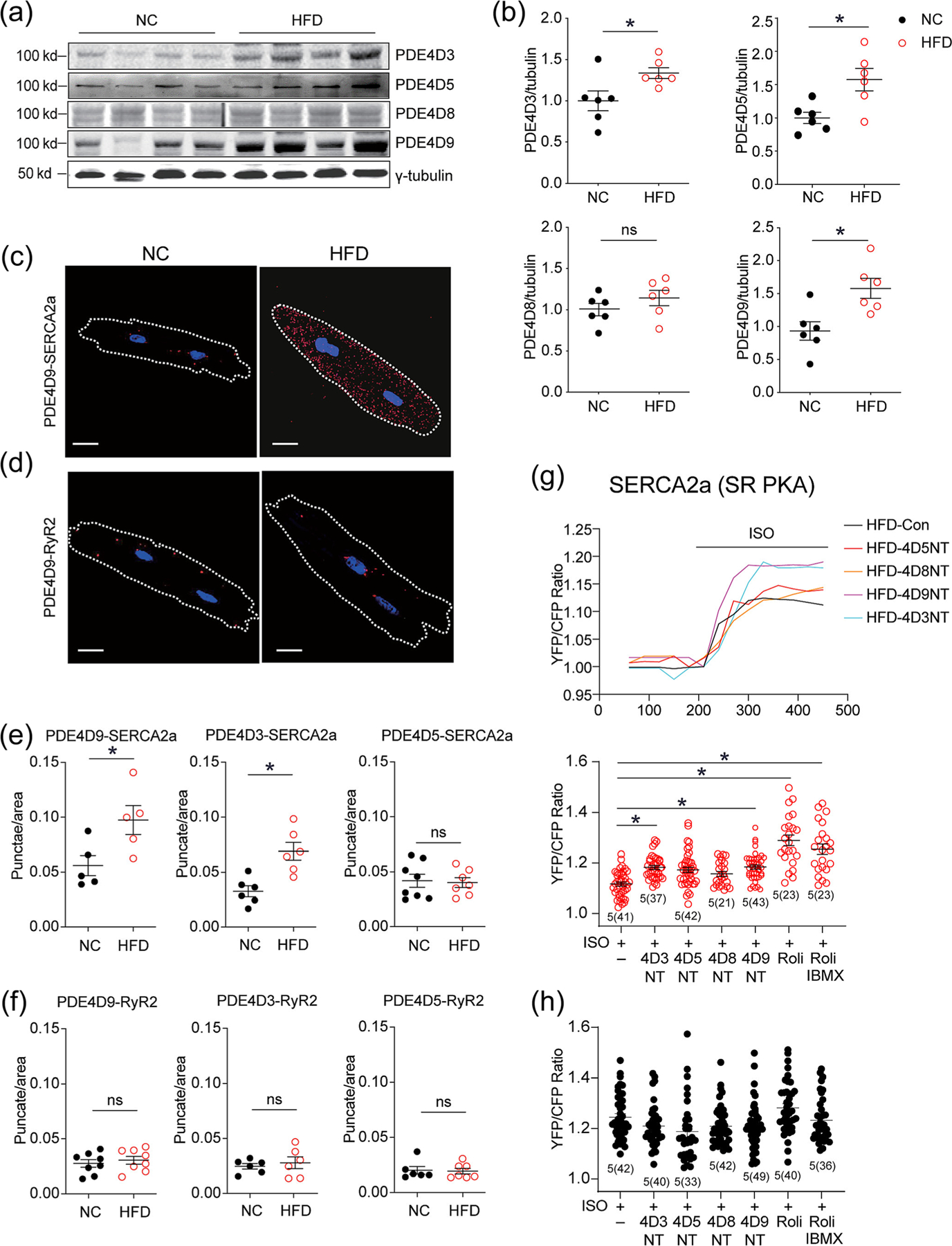
The upregulated PDE4D3 and PDE4D9 selectively associates with SERCA2a in high-fat diet (HFD)-fed myocytes. (a and b) Heart tissues from normal chow (NC) and HFD mice were subjected to western blot analysis of protein levels of PDE4D isoforms. The levels of protein expression were quantified in dot plots. (c–e) Isolated myocytes were subjected to PLA analysis with rabbit antibodies against PDE4D3, 4D5 and 4D9 together with an antibody agonist SERCA2a or RYR2, respectively. The PLA signals were detected with a Zeiss confocal microscope and quantified. (g and h) Isolated myocytes were infected with adenoviruses expressing SR AKAR3 biosensors. After treatment with membrane permeable dominant negative N-terminal (NT) PDE4D peptides (1 μM), myocytes were stimulated with isoproterenol (ISO) (100 nM), followed by addition of Roli (100 nM) and IBMX (100 mM) as indicated. The changes in FRET ratio were recorded. The maximal changes in the FRET ratio induced by ISO and other inhibitors were analysed and plotted. *P < 0.05 by Student *t* test in Figure 6b and e and by one-way ANOVA followed by Tukey’s test in Figure 6g.

Eleven isoforms of PDE4D (PDE4D1–11) have been reported. Among them, PDE4D3, PDE4D5, PDE4D8 and PDE4D9 are detected in hearts (Derici et al., 2019). They are long isoforms of PDE4D with unique N-terminal sequences, allowing them to interact with varying intracellular molecules (Kelly et al., 2014). In the hearts of HFD-fed mice, PDE4D3, 5 and 9 were elevated (Figures 6a, b, S8A). PLA assays were applied to determine whether PDE4D isoforms associate with SERCA2a in HFD myocytes. PDE4D3, PDE4D5 and PDE4D9 displayed moderate association with SERCA2a in NC myocytes, as indicated by puncta staining in PLA assays (Figures 6c–f and S8B,C). The numbers of PLA punctae were significantly increased in HFD cardiomyocytes when probing PDE4D9 or PDE4D3 together with SERCA2a (Figures 6c–f and S8C), indicating an elevated association between these PDE4D isoforms and the channel. As a control, PDE4D5’s association with SERCA2a did not change after HFD feeding (Figure 6e and f and S8C). Consistently, coimmunoprecipitation showed that PDE4 displayed an increased association with SERCA2a in HFD hearts relative to NC controls (Figure S8D). In contrast, none of these three PDE4D isoforms was found to be closely associated with RyR2 in myocytes from NC and HFD mice (Figure 6c–f and S8C).

PDE4D isoforms were then selectively targeted with membrane permeable dominant negative N-terminal (NT) peptides that displace the enzymes from binding to the partners, such as SERCA2a (De Arcangelis et al., 2009) (Figure S8E). Administration of inhibitory PDE4D3 and PDE4D9 NT peptides enhanced ISO-induced PKA activities at the SERCA2a domains in HFD myocytes (Figures 6g and h and S9A). PDE4D5 and 4D8 NT peptide administration did not affect the ISO-induced responses (Figures 6g and h and S9A). In contrast, none of the inhibitory NT peptides affected PKA activities at the SERCA2a domains in NC myocytes (Figures 6h and S9A). None of the inhibitory NT peptides affected PKA activities at the RyR2 domains in HFD myocytes either (Figure S9B).

### 3.5 | Inhibition of PDE4D3 or PDE4D9 restores β-adrenoceptor signalling at the SERCA2a nanodomains and E–C coupling in HFD-fed cardiomyocytes

**FIGURE 7:**
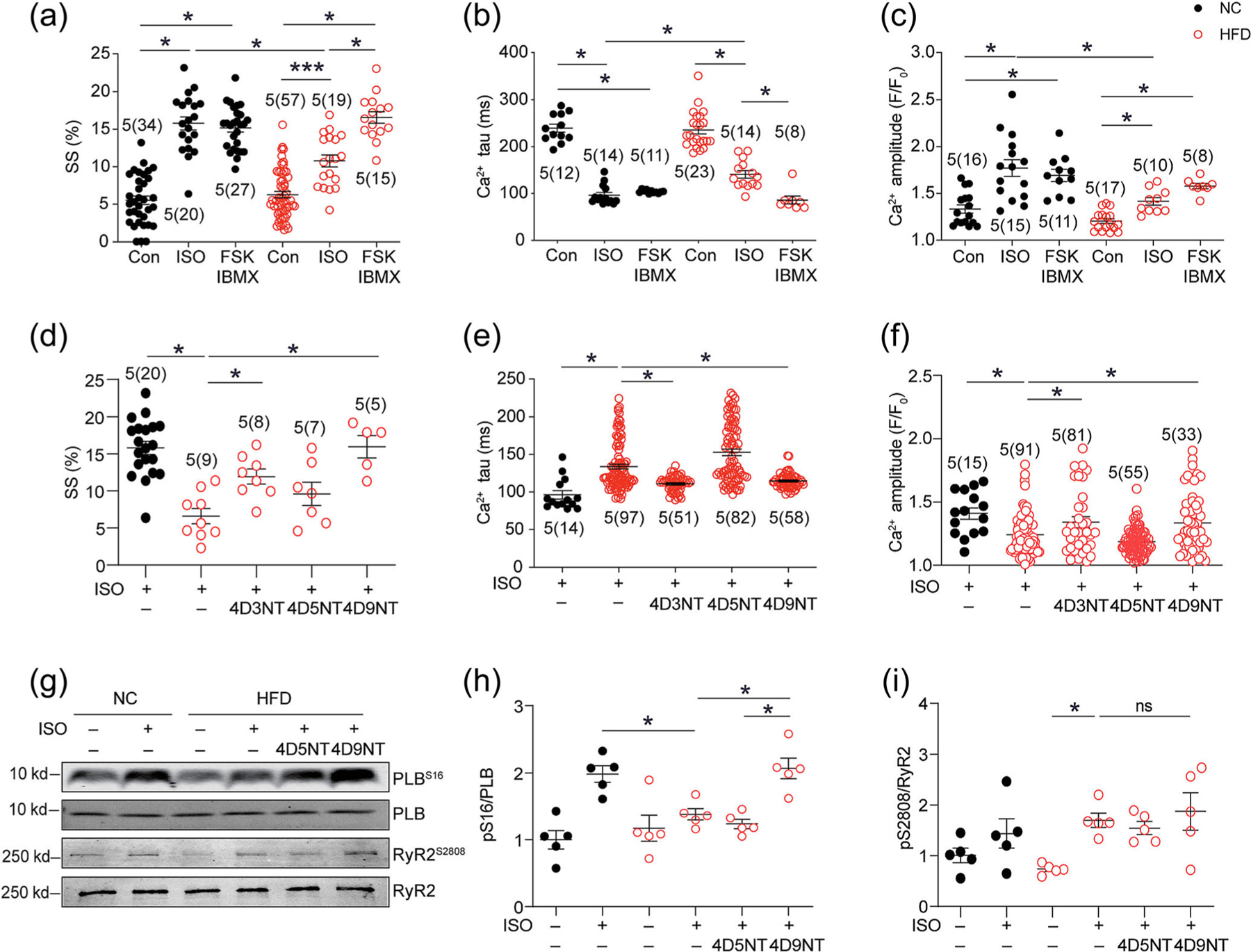
Inhibition of PDE4D3 or PDE4D9 restores subcellular β-adrenoceptor signalling and ECC in high-fat diet (HFD)-fed myocytes. Isolated myocytes were loaded with calcium dye Fluo-4AM and paced at 1 Hz. (a–c) Myocytes were treated with ISO, forskolin (FSK) (10 μM) and IBMX (100 μM) as indicated. The maximal changes in sarcomere shortening (SS) and calcium decay tau and transient amplitude were analysed and plotted. (d–f) Myocytes were pretreated with membrane permeable dominant negative N-terminal (NT) PDE4D isoform peptides (1 μM) before stimulation with ISO (100 nM). The maximal changes in sarcomere shortening and calcium decay tau and transient amplitude were plotted. (g–i) Cardiac myocytes were isolated from normal chow (NC) and HFD mice and treated with ISO (100 nM) in the presence of NT PDE4D isoform peptide (1 μM). Cell lysates were applied to western blots to detect the levels of total and phosphorylation of PLB at Ser16 and RyR2 at Ser2808. *P < 0.05 by two-way ANOVA followed by Tukey’s test. Con, control.

While ISO-induced responses in calcium handling and contractile function were impaired in HFD myocytes, co-administration of FSK and IBMX completely restored the speed of calcium decay tau together with increases in calcium transient peaks and contractile shortening (Figures 7a–c and S10A,B). These data indicate that HFD myocytes retain the capacity to increase E–C coupling even though the adrenergic-induced PKA signalling at the SERCA2a nanodomains is impaired. Both inhibitory PDE4D3 and PDE4D9 NT peptides markedly increased the calcium decline speeds under the treatment of ISO in diabetic myocytes (Figures 7e and S10A,B). Consequently, PDE4D9 and, to a lesser extent, PDE4D3 NT peptides effectively enhance ISO-induced calcium transient amplitude and contractile shortening in HFD myocytes (Figure 7d and f and S10A,B). As a control, the PDE4D5 NT peptide failed to improve calcium handling and contractile function in HFD myocytes (Figures 7d–f and S10A,B).

We further examined the impacts of PDE4D9 isoforms on PKA phosphorylation and PLB and calcium regulation in HFD myocytes. In agreement, the ISO-induced phosphorylation of PLB at Ser 16 but not Ser 2808 on RyR2 was significantly rescued by PDE4D9 NT peptide in HFD myocytes (Figure 7g–i). As a control, the PDE4D5 NT peptide did not affect the ISO responses (Figure 7g–i). These data further confirm that PDE4D3 and PDE4D9 selectively associate with SERCA2a in diabetic hearts and suppress the phosphorylation of PLB, leading to impaired SERCA2a function and diminished SR load in diabetic cardiomyopathy.

### 3.6 | Selectively inhibition or genetic deletion of PDE4D effectively ameliorates cardiac dysfunction in HFD-fed mice

**FIGURE 8:**
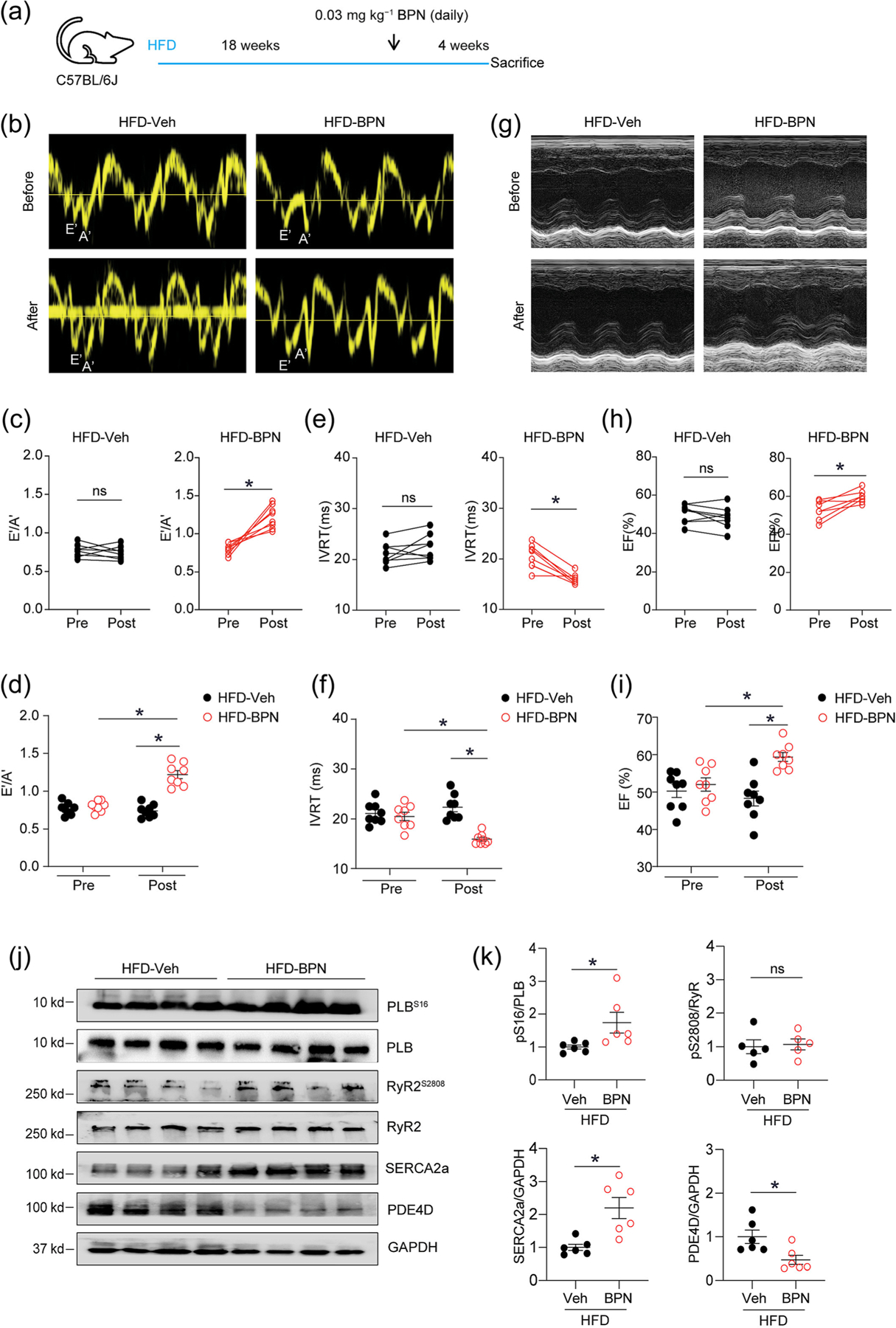
Selectively inhibition of PDE4D effectively ameliorates cardiac dysfunction of high-fat diet (HFD)-fed mice. (a) Diagram shows that WT mice were fed with HFD for 4.5 months and then treated with saline or BPN (0.03 mg kg^−1^) for 4 weeks. (b–i) Echocardiography was used to detect diastolic function parameters E′/A′ and isovolumic relaxation time (IVRT) and systolic function parameter ejection fraction (EF) in HFD mice after treatments. (j and k) After treatments, heart tissues from HFD mice were harvested for western blot analysis to detect the expression of PLB, SERCA2a, RyR2 and GAPDH, as well as phosphorylation of PLB at Ser16 and phosphorylation of RyR2 at Ser2808. The western blots were quantified and presented in dot plots. *P < 0.05 by one-way ANOVA followed by Tukey’s test and Student *t* test.

Although targeting the elevated expression of PDE4D by the PDE4 inhibitor was effective in treating DCM in the HFD-fed mice, the PDE4 inhibitor has low therapeutic index because of systemic side effects including nausea, emesis, diarrhoea and headache (Phillips, 2020). Subsequently, the PDE4D-specific allosteric inhibitor BPN14770, which targets long isoforms, was used to treat HFD-fed induced mice DCM (Berry-Kravis et al., 2021). Mice fed with HFD for 4 months displayed diastolic cardiac dysfunction manifesting with an E′/A′ ratio less than 1 and a prolonged IVRT. HFD mice treated with BPN14770 displayed reduced body weights as well as heart, liver and visceral fat weights relative to saline treated controls (Figure S11F–I). Therapy with BPN14770 also slightly improved glucose and insulin sensitivities with reduced plasma glucose levels in HFD mice (Figure S11A–E). Compared with vehicle-treated DCM mice, BPN14770 therapy for 4 weeks effectively restored the E′/A′ ratio and reduced the IVRT duration (Figure 8a–f). Of note, the EF percentage was substantially improved upon BPN14770 treatment relative to the vehicle group (Figure 8g–i and Table S3), indicating that not only diastolic dysfunction but also systolic dysfunction could be rescued by BPN14770 administration. Furthermore, the phosphorylation of PLB at Ser16 site but not Ser 2808 on RyR2 was upregulated in myocardium from BPN14770 treated DCM mice (Figure 8j and k). BPN14770 treatment increased the expression of SERCA2a while reducing the expression of PDE4D in HFD mice (Figure 8j and k). In addition, Masson’s staining revealed definite cardiac fibrosis in HFD-fed mice. BPN14770 significantly ameliorated the fraction of interstitial and perivascular fibrosis (Figure S11J–L), and it also reduced the area of cardiomyocytes in DCM mice compared with vehicle-treated DCM mice (Figure S11M–N).

Similarly, deletion of PDE4D displayed preserved heart functional parameters including E′/A′, IVRT, EF and the phosphorylation of PLB at Ser 16 in hearts after HFD feeding (Figure S12A–C). Taken together, selective inhibition of PDE4D could improve both diastolic and systolic heart function of DCM mice by restoring E–C coupling of myocytes and leads to notable alleviation in the pathomorphological changes of the myocardium as well, indicating that the PDE4D inhibitor may be superior to PDE4 inhibition in treating HFD-fed induce DCM.

## 4 | DISCUSSION

Despite the prominent expression and function of PDE4 enzymes in hearts, clinical usage of PDE4 inhibitors has yet to be fruitful because of an incomplete understanding of the complex expression and regulation of individual isoforms in cardiac diseases. Pan PDE4 inhibitors have been utilised to treat airway inflammation by inhibiting the activation of immunocytes (Chong et al., 2017; Wedzicha et al., 2016). However, the implementation of pan-PDE4 inhibitors has been limited because they often have adverse reactions, such as nausea, vomiting and a higher risk of arrhythmia because of the extensive distribution of different PDE4 isoforms in a variety of tissues (Eschenhagen, 2013). Therefore, it is essential to identify the predominantly elevated PDE4D isoforms in DCM cardiomyocytes (Wang et al., 2017; Xu, Fu, et al., 2022), which may present promising targets for developing safe and effective therapeutic drugs. In this study, our data show that the isoforms PDE4D3 and PDE4D9 are selectively upregulated in DCM hearts. PDE4D3 and PDE4D9 isoforms specifically target the SERCA2a nanodomains, leading to impaired PKA phosphorylation of PLB, calcium decay tau, and SR calcium load and contributing to impaired myocyte contractile function in DCM. Therapies with PDE4D inhibitor BPN14770 restored PKA phosphorylation of PLB and cardiac contractile function. These data indicate that the upregulation of PDE4D3 and PDE4D9 isoforms is a significant pathological feature of DCM; specific targeting of these isozymes may restore cardiac function without increasing the risk of arrhythmias, which could be a potential target for the treatment of DCM.

Studies show that PDE4A and PDE4D are significantly reduced in the hearts of patients with idiopathic dilated cardiomyopathy (Richter et al., 2011). Additional reports suggest that the relative activity and protein expression of PDE4A and PDE4B are reduced in hypertrophic cardiomyocytes but do not affect the protein expression of PDE4D (Abi-Gerges et al., 2009). The genes of the PDE4 isoforms share similarities in that they consist of similar and evolutionarily conserved exons encoding the enzyme catalytic domain and two regulatory domains (upstream conserved region 1 [UCR1] and upstream conserved region 2 [UCR2]) (Paes et al., 2021); and they differ in that the PDE4 genes contain different alternative promoters that generate different transcript variants by combining unique exons and splicing mechanisms. Each promoter contains different transcriptional response elements (cAMP-response element binding protein [CREB], melanocyte inducing transcription factor [MITF] or activating transcription factor 4 [ATF4]) (Khaled et al., 2010; Li et al., 2021; Sheppard et al., 2014) that are associated with the transcriptional regulation of multiple signalling pathways. These complex transcriptional regulatory mechanisms likely play a role in upregulating the expression of different PDE4 isoforms in response to pathological stimuli.

SERCA2a is a key molecule in the regulation of cardiac contraction and relaxation. The expression and activity of SERCA2a are generally reduced in heart failure patients. Researchers have carried out extensive basic studies and clinical trials (CUPID studies) on restoring SERCA2a expression and activities directly, aiming to recover cardiac function of heart failure patients (Chelu & Li, 2018; del Monte et al., 2002; Greenberg et al., 2016; Hajjar et al., 2008). Researchers have also reported in preliminary results that targeting the SUMCA-1 gene improved the cardiac function of mice with heart failure via regulating SERCA2a activity (Kho et al., 2011; Tilemann et al., 2013). SERCA2a is also directly regulated by PLB via protein–protein interaction. PLB binds to and inhibits SERCA2a in a resting form. Once PLB is phosphorylated by PKA at Ser 16 or CaMKII at Thr 17, it is removed from the binding site of SERCA2a and triggers SERCA2a activation (Smeazzetto et al., 2017). Therefore, strategies that displace PLB from binding to SERCA2a, such as a newly identified DWORF peptide, hold promise in restoring SERCA2a function in cardiac disease (D. M. Anderson et al., 2015; Nelson et al., 2016).

Moreover, the local PKA activity at the SERCA2a complex is often selectively reduced in cardiac diseases, contributing to reduced PLB phosphorylation and SERCA2a function (Ahmad et al., 2015; Liu et al., 2015; Nicolaou et al., 2009). The critical regulators of the PLB/SERCA2a interaction include phosphatase 1 isoforms and PDEs (Ahmad et al., 2015; Liu et al., 2015; Nicolaou et al., 2009). Compared to extensive studies on phosphatases, little is known about how PDEs are involved in the pathological remodelling of cAMP signals and PKA activities at the SERCA2a complex. Interestingly, we have recently demonstrated an intracellular β_1_-adrenoceptor pool associated with SERCA2a on the SR (Y. Wang et al., 2021). Previous studies show that the β_1_-adrenoceptor-induced cellular signalling is highly regulated by the receptor-associated PDE4D isoforms (De Arcangelis et al., 2009; Fu, Kim, et al., 2014; Richter et al., 2008), raising the possibility that PDE4D isoforms may be directly involved in regulating PKA phosphorylation of PLB at the SERCA2a complex in cardiac diseases. In the present study, we have identified that PDE4D3 and PDE4D9 are selectively upregulated in diabetic myocytes, where these isoforms specifically diminish the local cAMP–PKA activities associated with SERCA2a but not RyR2 nanodomains. These data are consistent with the minimal change or upregulation of PKA activities at the RyR2 complex in human heart failure (Ahmad et al., 2015; Liu et al., 2015; Nicolaou et al., 2009). The higher levels of PDE4D3/9 are accompanied by reduced levels of phosphorylation at the Ser 16 of PLB and calcium decay tau, contributing to suppressed SR calcium load and reduced calcium transient amplitude and contractile shortening.

Of note, the elevated CaMKII phosphorylation of PLB at Thr 17 in the HFD hearts may compensates the reduced PKA phosphorylation of PLB, leading to a normal relaxation at basal state in DCM myocytes. However, the impacts of PDE4D on the phosphorylation of PLB and calcium handling can be revealed with PDE4 inhibitor or after beta adrenergic stimulation. Another study using db/db mice has revealed that local depletion of PDE4 from SERCA2a microdomains might act as a compensatory mechanism to prevent diastolic function decline (Lai et al., 2024). Although evidence has shown that heart failure patients with preserved ejection fraction display distinct heterogeneity (Rosch et al., 2022), studies from db/db mice also display distinct alteration of calcium handling when compared those from HFD feeding (Mira Hernandez et al., 2024). These studies indicate that the models may significantly impact the molecular and structural remodelling in the diabetic heart.

Inhibition of the PDE4D3 and 4D9 isoforms with membrane-permeable dominant-negative NT peptides restores PKA activity, phosphorylation of PLB, calcium handling and contractility in diabetic myocytes (Figure S6A,B). Compared to previous reports (Abi-Gerges et al., 2009; Richter et al., 2011), it is notable that the elevation of these PDE4D isoforms is only observed in DCM. While these observations remain to be validated in human patients, the observations hold a promise that these PDE4D isoforms can be selectively targeted in the treatment of DCM. Meanwhile, previous reports show that PDE4D3 also binds to RyR2, and global deletion of PDE4D leads to calcium leakage through RyR2 and heart failure in ageing mice (Lehnart et al., 2005). However, our observations are distinct from these previous reports. (1) We observed increased PDE4D isoform expression in diabetic hearts, whereas the gene is downregulated in MI hearts, in which further inhibition of PDE4D may exacerbate the cardiac dysfunction, and (2) inhibition of PDE4D could have distinct long-term outcomes when compared to complete gene deletion (Lehnart et al., 2005). Further studies are needed to titrate the dose impacts of genetic reduction of PDE4D, such as heterozygous deletion of PDE4D, in different cardiac diseases. Moreover, our studies show that selective blocking of the association of PDE4D9 from SERCA2a may distinctly restore the activities of SERCA2a while sparing the detrimental effects on other proteins, such as RyR2 in diabetic myocytes, which may present an effective strategy to improve the heart function of DCM patients.

PDE inhibitors will likely exert secondary effects in vivo. For instance, BPN14770 treatment ameliorates glucose metabolism and insulin sensitivity, with reducing body, visceral fat, and liver weights. However, the data with PDE4D knockout mice and the data from isolated myocytes supports that PDE inhibitors have a direct impact on myocytes in the heart. In conclusion, our data reveal an upregulation of PDE4D3/9 isoforms selectively localised to the SERCA2a complex in cardiomyocytes. This unique pathological change sheds light on the novel pathogenesis of DCM and affords promising pathways for disease-specific DCM treatment through either neutralising the specific PDE4D isoforms or disrupting the PDE4D3/9-SERCA2a association.

## Supplementary Material

Online Figures and Tables

Additional supporting information can be found online in the Supporting Information section at the end of this article.

## Data Availability

The data that support the findings of this study are available from the corresponding author upon reasonable request.

## What is already known?

- Hyperinsulinaemia upregulates PDE4D expression in diabetic cardiomyocytes.
- SERCA2a activity is downregulated in the diabetic heart, leading to impaired heart function.

## What does this study add?

- The upregulated PDE4D isoforms bind to and inhibit SERCA2a but not RyR2 in diabetic cardiomyocytes.
- Selectively inhibiting PDE4D effectively restores cardiac function in diabetic cardiomyopathy.

## What is the clinical significance?

- Targeting PDE4D isoforms may be an effective strategy to treat diabetic cardiomyopathy.

## Abbreviations:

A′: the mitral valve annulus during late diastole
AVM: adult left ventricular myocytes
CFP: cyan fluorescent protein
Cilo: cilostamide
DCM: diabetic cardiomyopathy
E′: the mitral valve annulus during early diastole
ECC: excitation–contraction coupling
EF: ejection fraction
EHNA: (erythro-9-(2-hydroxy-3-nonyl) adenine
FRET: fluorescence resonance energy transfer
FS: fractional shortening
FSK: forskolin
HFD: high-fat diet
IACUC: Institutional Animal Care and Use Committees
IBMX: 3-isobutyl-1-methylxanthine
ISO: isoproterenol
IVRT: isovolumic relaxation time
MI: myocardial infarction
MITF: melanocyte inducing transcription factor
NC: normal chow
NT: N-terminal
PLA: proximity ligation assay
PLB: phospholamban
PM: plasma membrane
Rofl: roflumilast
Roli: rolipram
SERCA2a: sarcoplasmic reticulum Ca^2+^-ATPase
Sild: sildenafil
SR: sarcoplasmic reticulum
SS: sarcomere shortening
TnI: troponin I
YFP: yellow fluorescent protein
