# Supplementary material for: Inhibition of the upregulated phosphodiesterase 4D isoforms improves SERCA2a function in diabetic cardiomyopathy
Source: Br J Pharmacol. Author manuscript; Available in PMC 2025 Jul 3. (PMC12225536; doi:10.1111/bph.17411)
Supplement: Online Figures and Tables [file NIHMS2093089-supplement-Online_Figures_and_Tables.docx]

**Title:** **Inhibition of the upregulated phosphodiesterase 4D isoforms improves SERCA2a function in diabetic cardiomyopathy**

**Short title: PDE4D3/9 inhibits SERCA2a in diabetic hearts**

**Authors:** Zhenduo Zhu^1, #^, Qiuyun Guan^1, #^, Bing Xu^2,3^, Sherif Bahriz^2^, Ao Shen^4^, Toni M. West^2^, Yu Zhang^4^, Bingqing Deng^5^, Wei Wei^1^, Yongsheng Han^6^, Qingtong Wang^1,2,*^ and Yang K. Xiang^2,3, *^


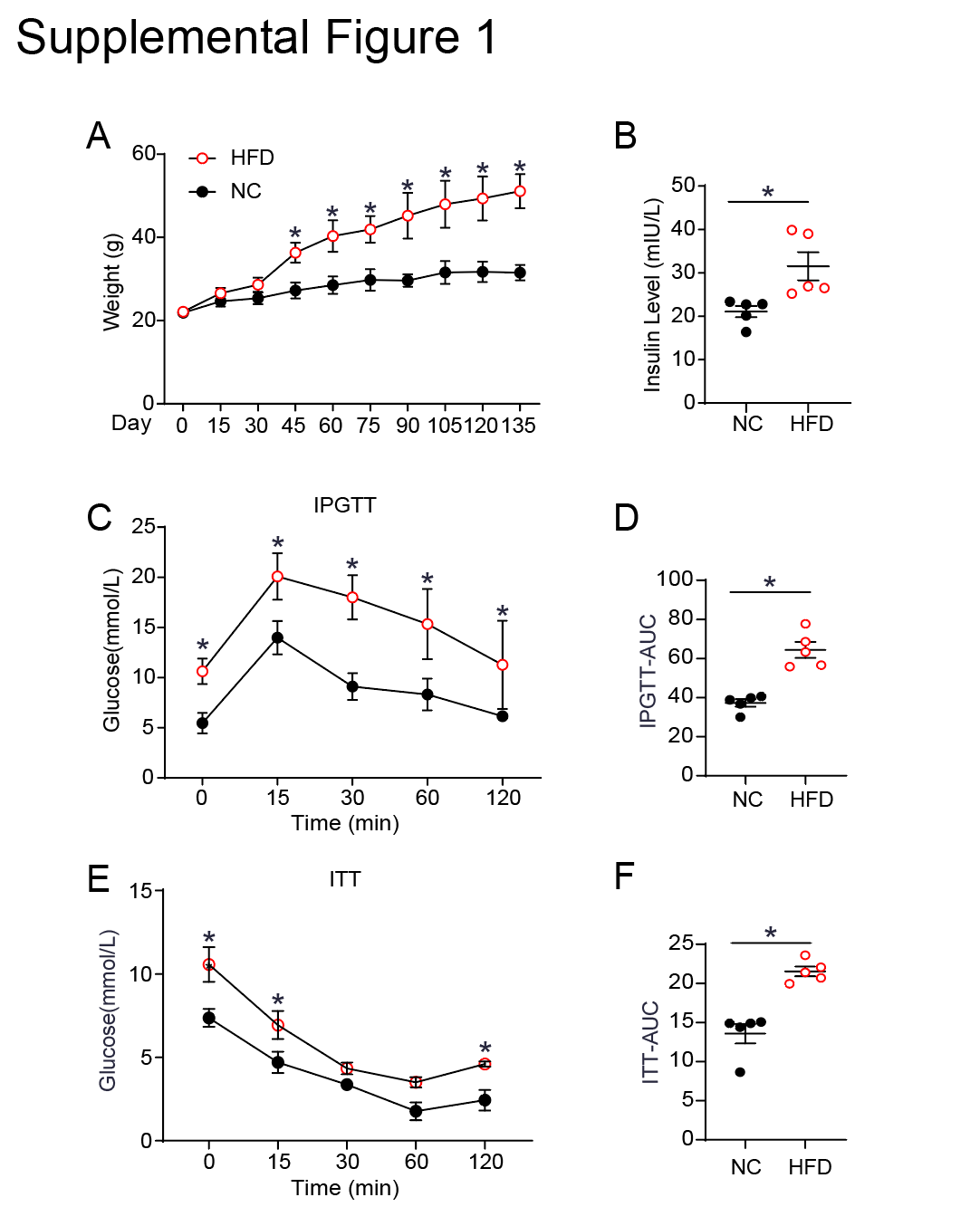


**Fig S1.** Characterization of NC and HFD mice. A and B) Weight and insulin levels of NC and HFD mice after 18 weeks of feeding. C and D) IPGTT and ITT of NC and HFD mice after 18 weeks of feeding. * *p* < 0.05 by two-way ANOVA followed by Tukey’s test in panel A, C and E, and by student *t* test in panel B, D, and F.


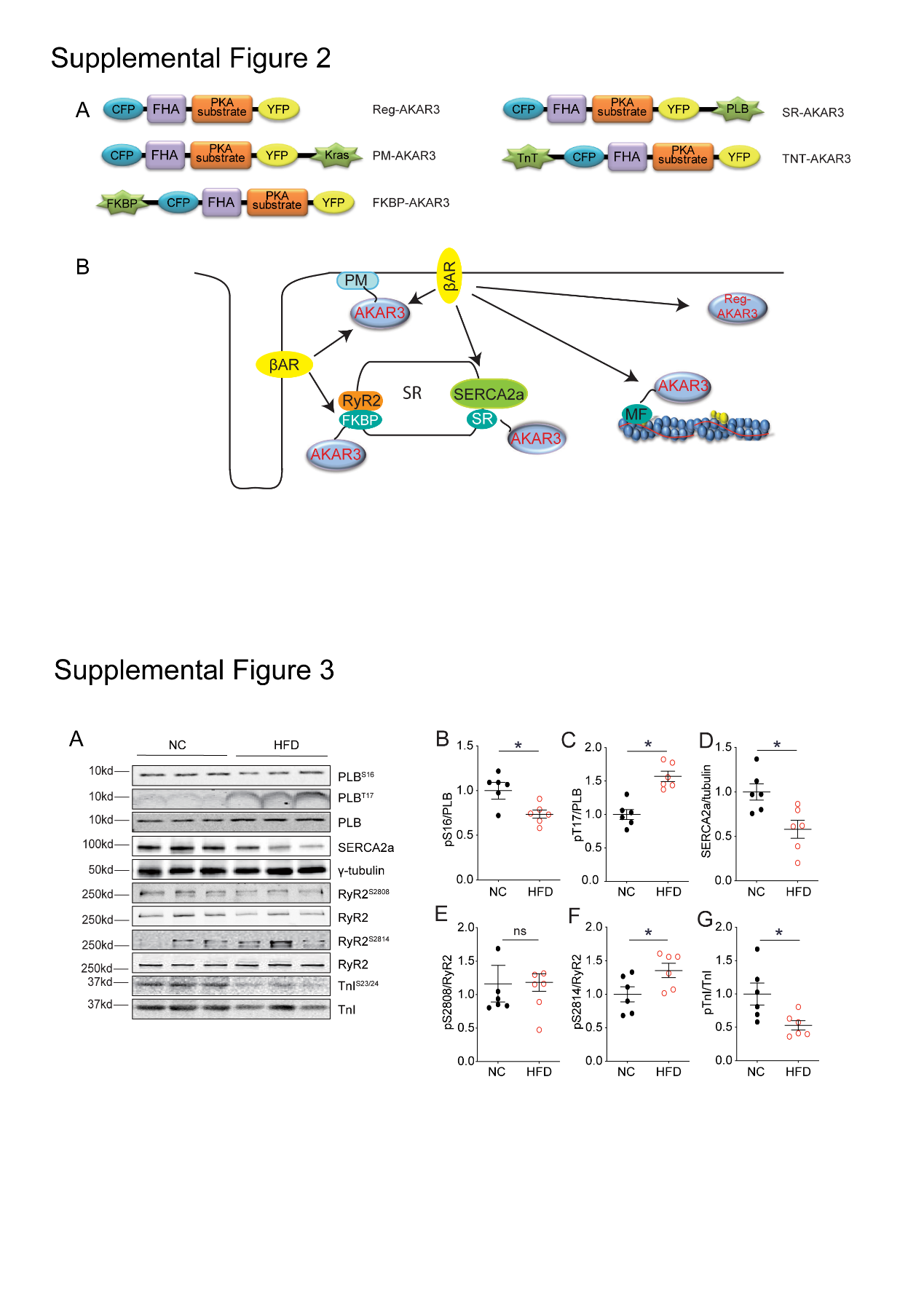
 **Fig S2**. A Structural illustration of a series of AKAR3 with specific domains to target to subcellular compartments. Kras, Kirsten rat sarcoma virus; FKBP, FK506 binding protein, PLB, phospholamban, TnT, troponin T. B) Cartoon depicts which nanodomains the PKA biosensors of AKAR3 target on the subcellular compartments, PM-AKAR3 targeted to the plasma membrane, FKBP-AKAR3 targeted to the RyR2 nanodomains; SR-AKAR3 targeted to the SERCA2a nanodomains, and TnT-AKAR3 targeted to the myofilaments.


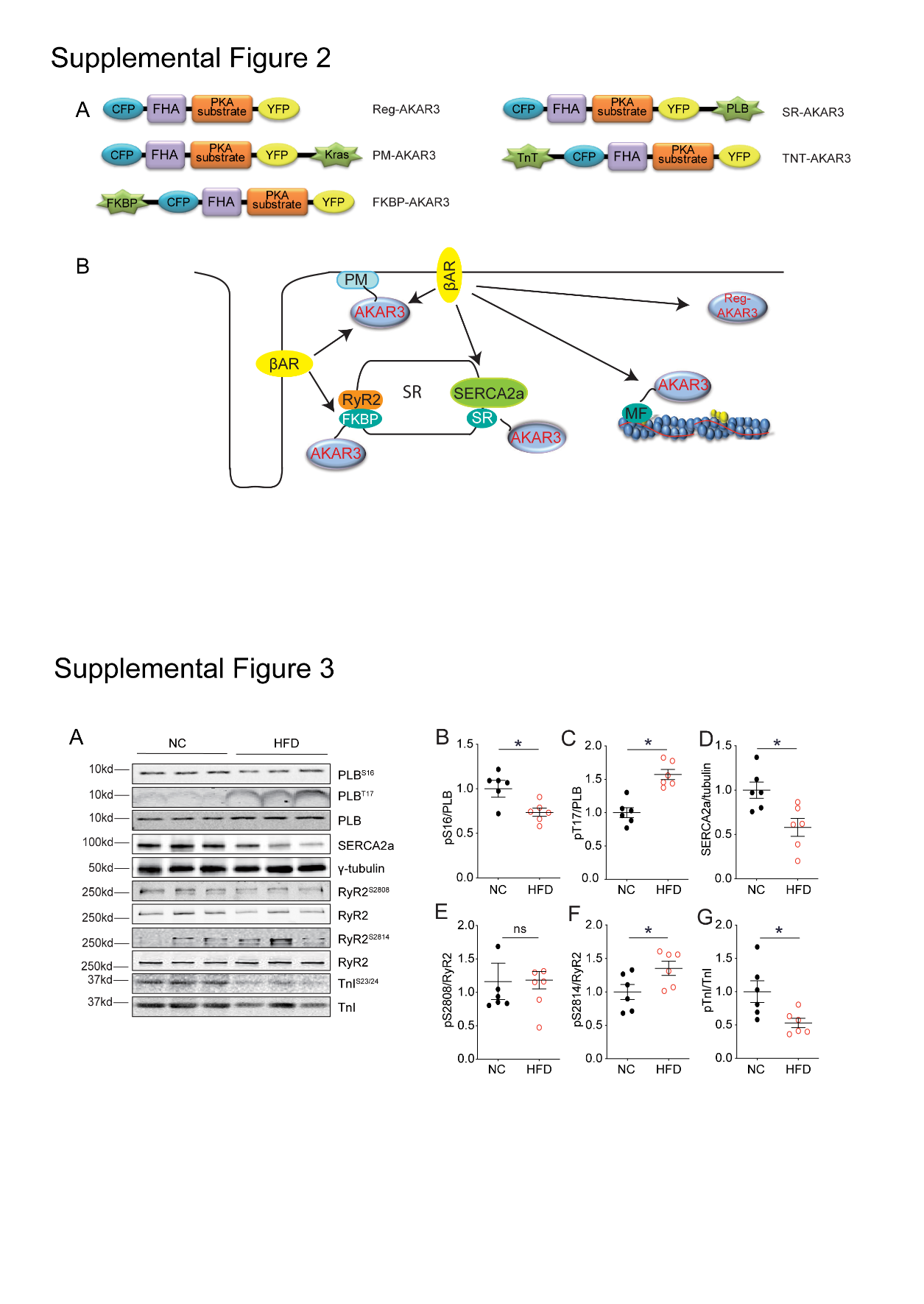


**Fig. S3**. A-G) WT mice were fed with HFD or NC diet for 4.5 months. Heart tissues were harvested for western blot analysis to detect the expression of PLB, SERCA2a, RyR2, TnI, and γ-tubulin, as well as phosphorylation of PLB at Ser16 and Thr17, phosphorylation of RyR2 at Ser2808 and Ser2814, and phosphorylation of TnI at Ser23/24. The quantification of western blots is presented in dot plots. * *p* < 0.05 by student *t* test.


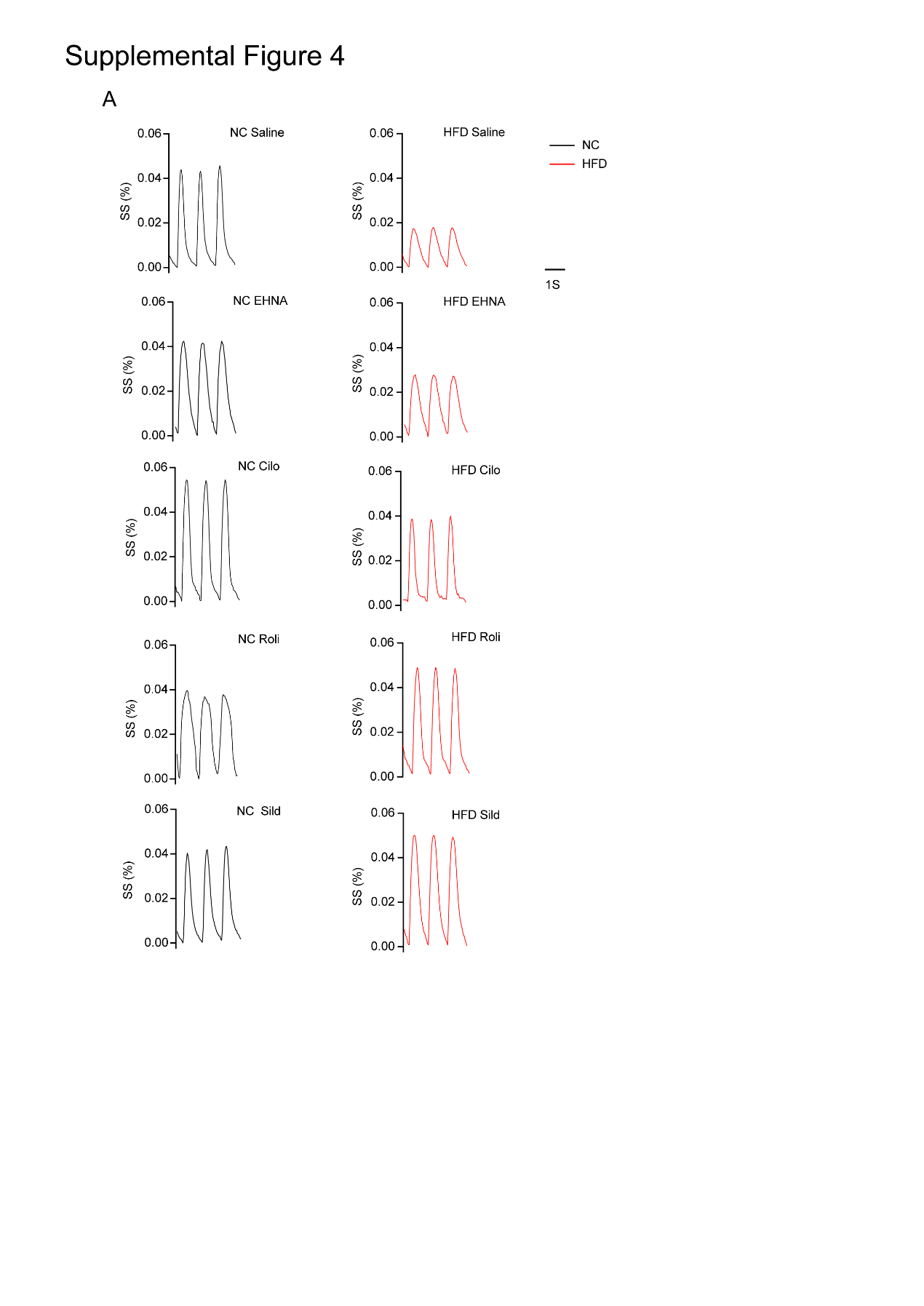


**Fig. S4**. Myocytes were loaded with calcium dye Fluo-4 AM and paced at 1 Hz. Calcium signals and contractility were recorded after addition of ENHA (10 μM), Cilo (1 μM), Roli (10 μM), and Sild (1 μM) as indicated. Representative contractile shortening traces were shown.

**
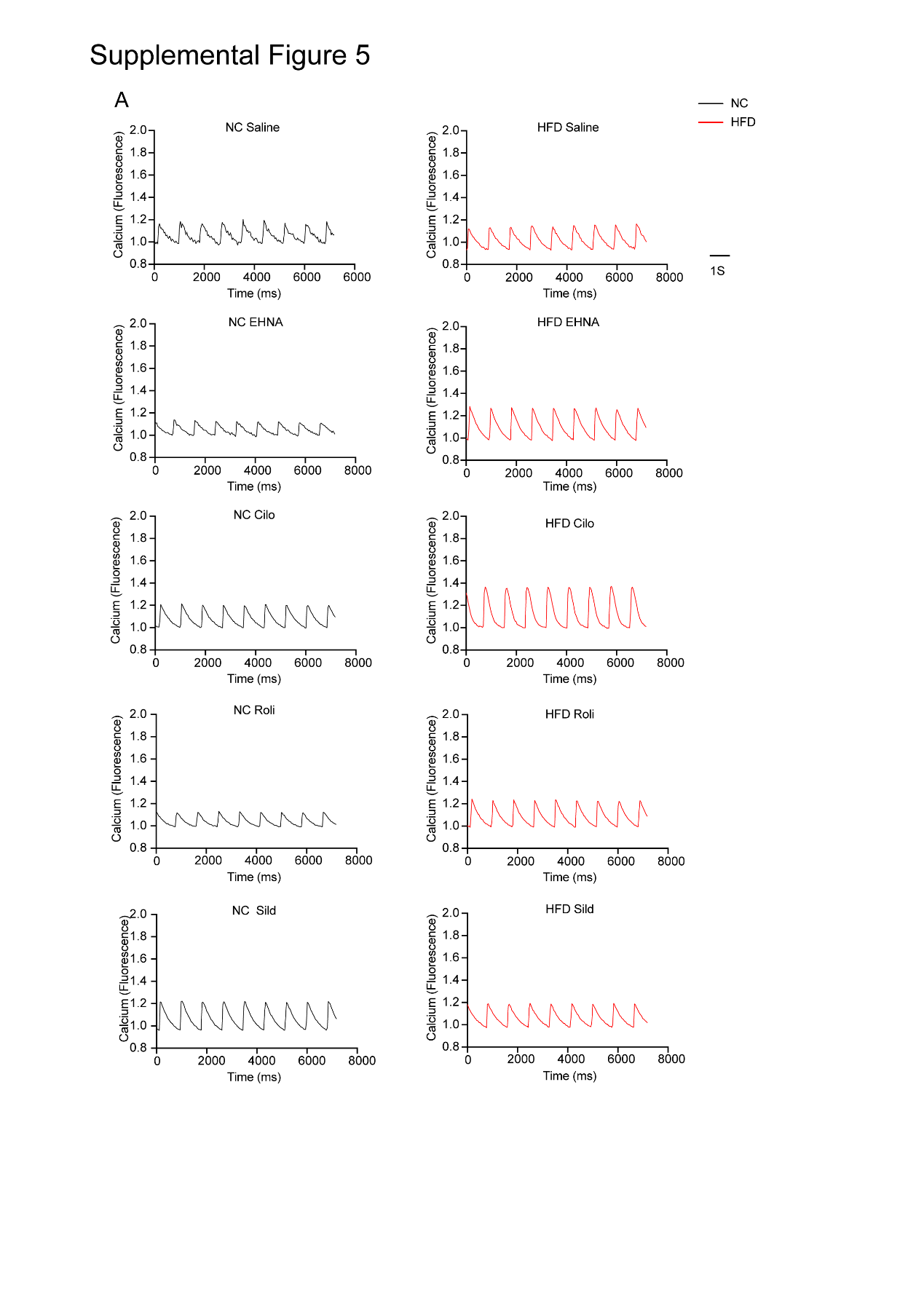
Fig. S5**. myocytes were loaded with calcium dye Fluo-4 AM and paced at 1 Hz. Calcium signals and contractility were recorded after addition of ENHA (10 μM), Cilo (1 μM), Roli (10 μM), and Sild (1 μM) as indicated. Representative calcium transient traces were shown.


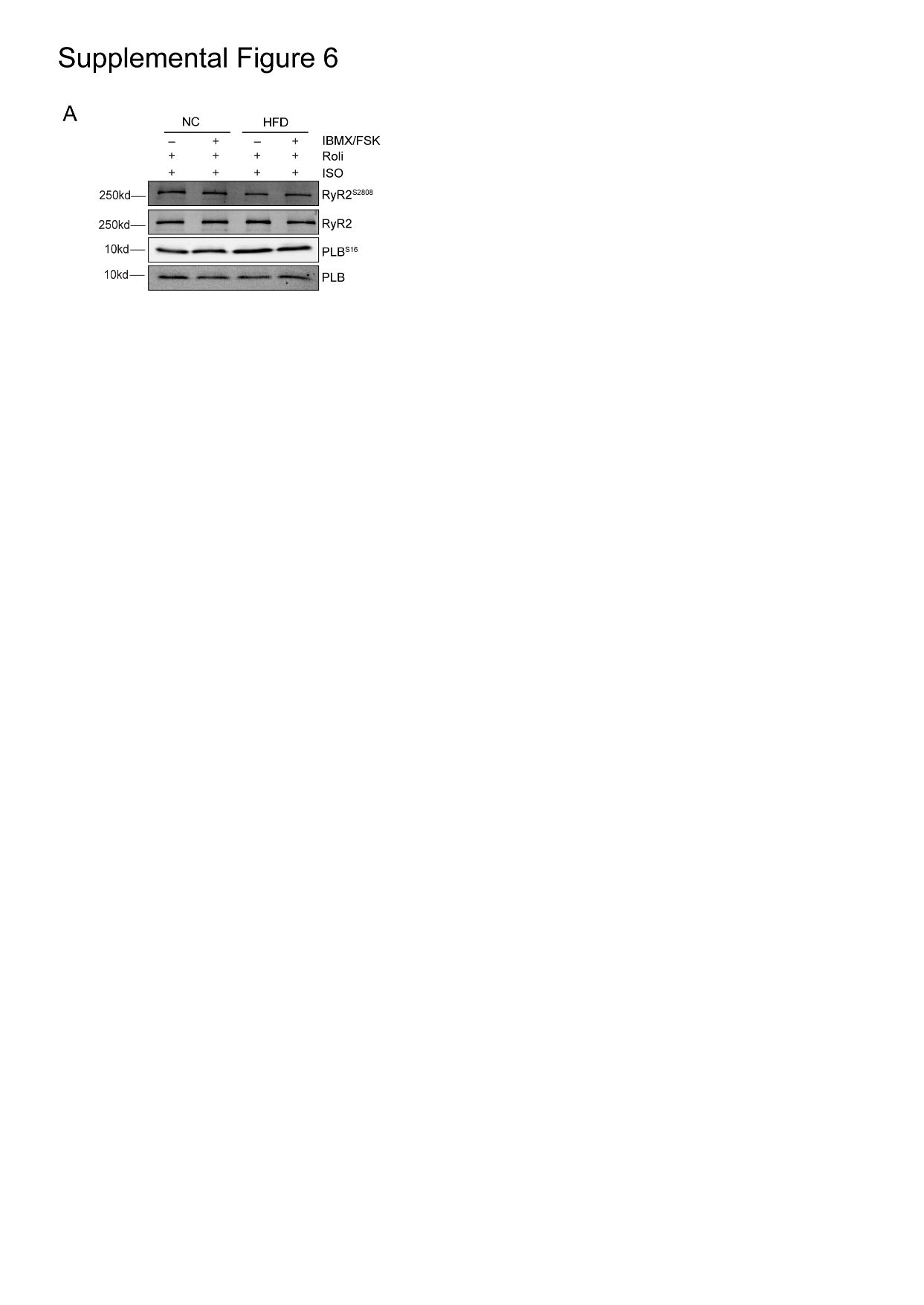
 **Fig. S6** Cardiac myocytes isolated from NC or HFD mice were treated with ISO (100 nM), Roli (100 nM), and IBMX (10 μM) as indicated. Representative western blots show the levels of phosphorylation of PLB at Ser16 and RyR2 at Ser2808 and total PLB and RyR2.


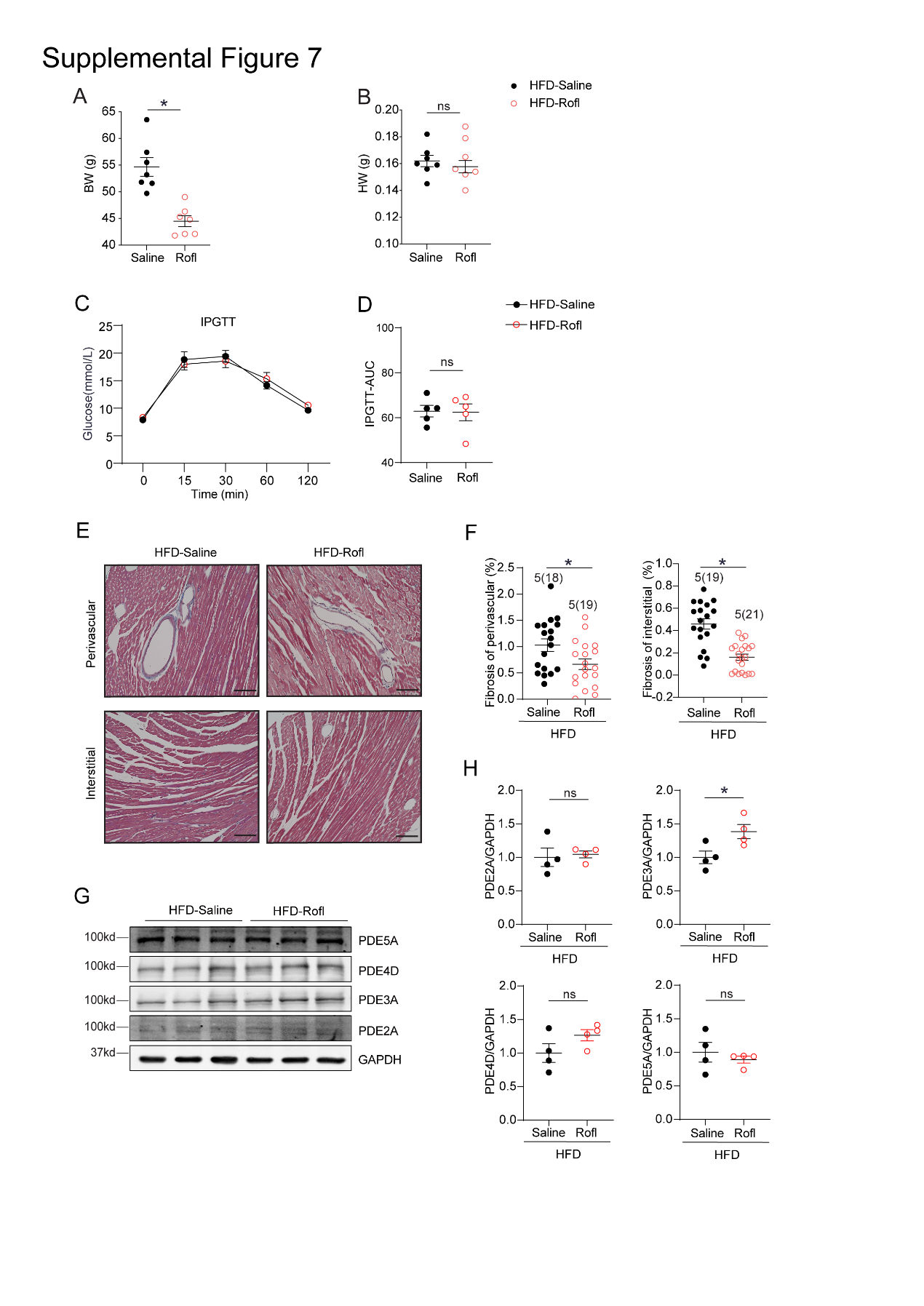


**Fig. S7**. WT mice were fed with HFD diet for 4.5 months before treatment with Saline or the PDE4 inhibitor Rofl for 4 weeks. A and B) The body weight and heart weight were measured and plotted. C and D) Mice underwent IPGTT test, the time couses and AUC of blood glucose levels after intraperitoneal glucose injection were determined. E and F) The cardiac fibrosis was detected by Masson's staining. The perivascular and interstitial cardiac fibrosis were analyzed in saline and Rofl-treated mice. Scale bar = 50 µm. G-H) The expression levels of PDE2A, 3A, 4, and 5A proteins in cardiac tissues after saline and Rofl treatment of HFD mice were detected and quantified in western blot. * *p* < 0.05 by two-way ANOVA followed by Tukey’s test in panel C, and by student *t* test in other panels.


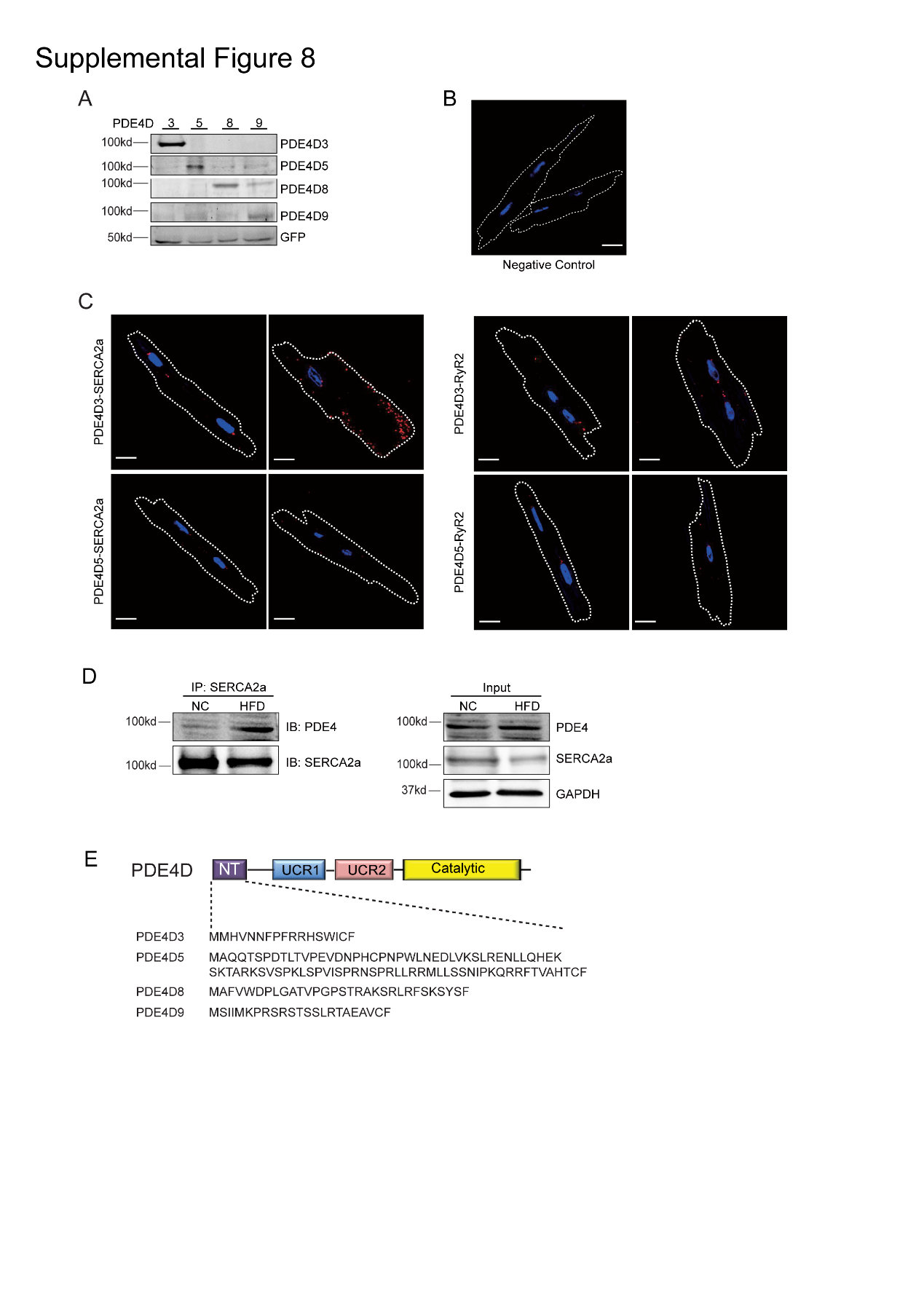


**Fig. S8**. A) The specificity of antibodies to PDE4D3, 5, 8, and 9 were confirmed with overexpressed PDE4D isoforms in HEK293 cells. B) Negative control for PLA experiments was performed with mouse and rabbit IgG antibodies in NC myocytes. C) Myocytes isolated from NC and HFD mice were subjected to PLA analysis with rabbit antibodies against PDE4D isoforms together with a mouse monoclonal antibody agonist SERCA2a or RYR2, respectively. D) SERCA2a were immunoprecipiated with mouse monoclonal antibody from NC and HFD heart lysates. Representative images show SERCA2a and PDE4 immunoprecipitated with SERCA2a antibodies in NC and HFD hearts. E) Cartoon depicts the unique N-terminal sequence of PDE4D3, 5, 8, and 9 isoforms.


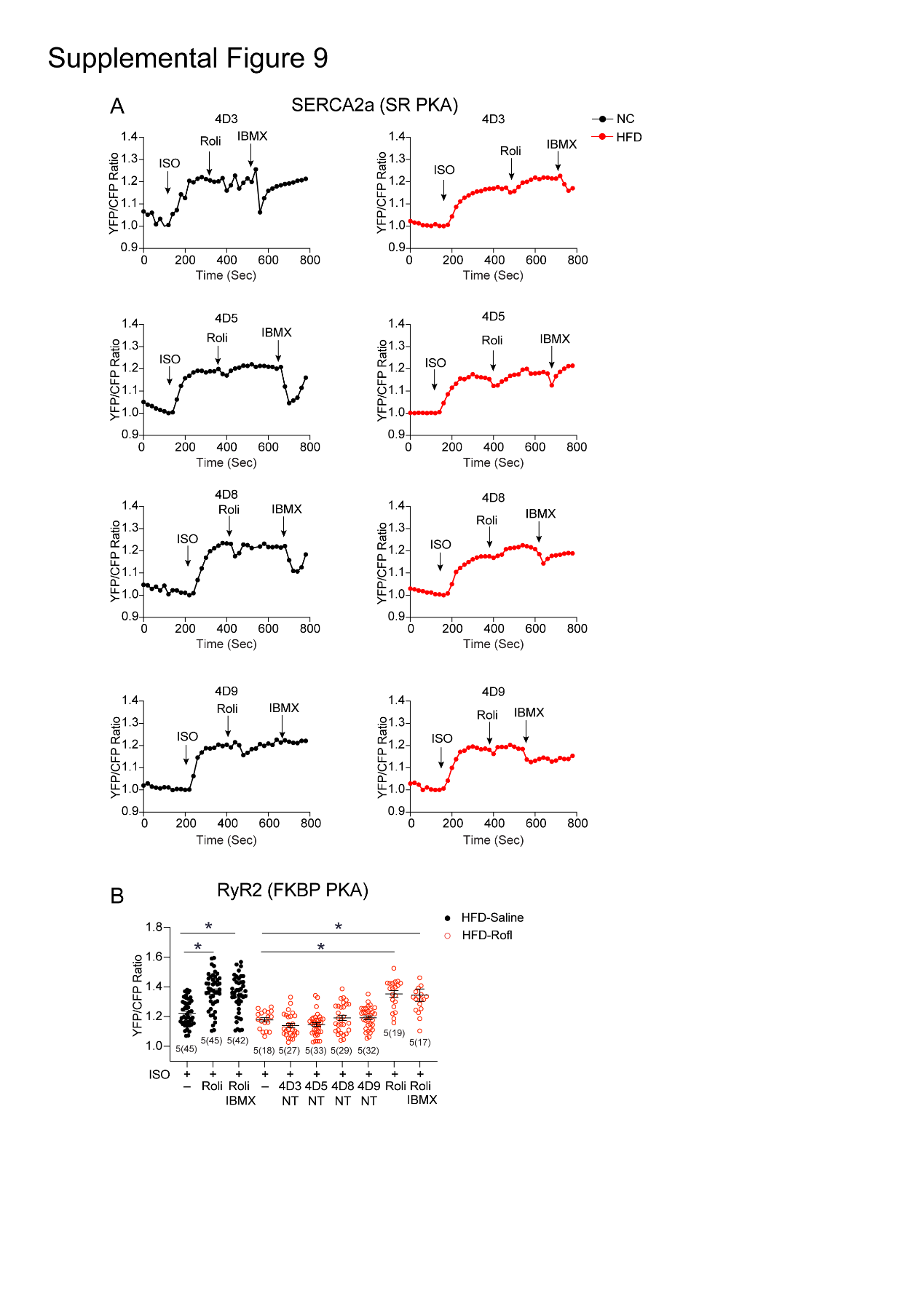


Fig. S9. A) Myocytes were isolated and infected with adenoviruses expressing SR AKAR3 biosensors. After treatment with membrane permeable dominant negative N-termimal (NT) PDE4D peptides (1 μM), the FRET ratio was recorded after addition of ISO (100 nM) followed by Roli and IBMX as indicated. change in the FRET trace were plotted. B) Myocytes were isolated and infected with adenoviruses expressing FKBP AKAR3 biosensors. After treatment with membrane permeable dominant negative (NT) PDE4D peptides (1 μM), Roli, and IBMX, myocytes will stimulated with ISO (100 nM). The maximal increases in the FRET ratio were plotted. * *p* < 0.05 by two-way ANOVA followed by Tukey’s test.


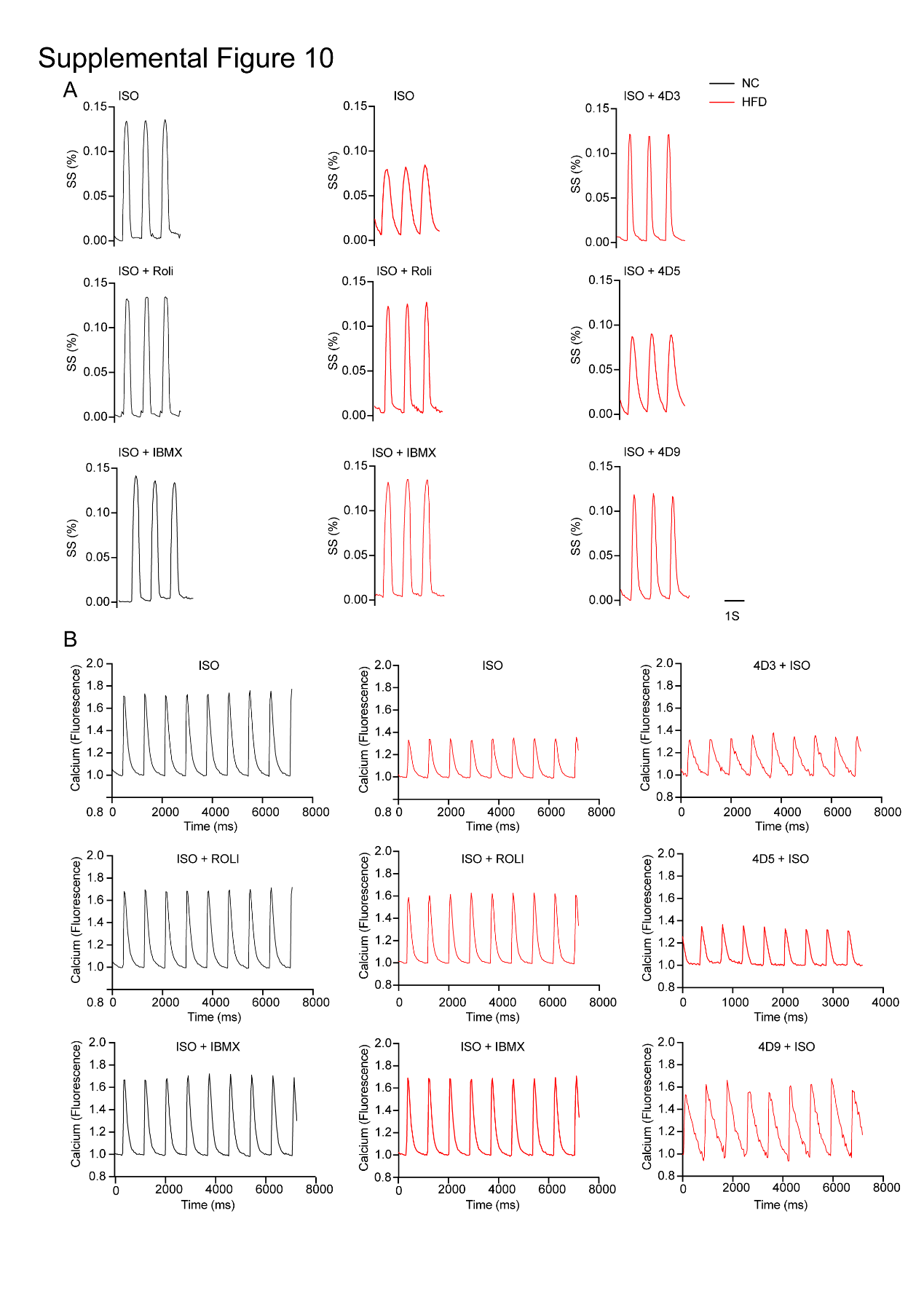


**Fig 10.** Myocytes were loaded with calcium dye Fluo-4 AM and paced at 1 Hz. Calcium signals and contractility were recorded after addition of ISO (100 nM), Roli (100 nM), and IBMX (10 μM) as indicated. Representative contractile shortening (A) and calcium transient (B) traces were shown.


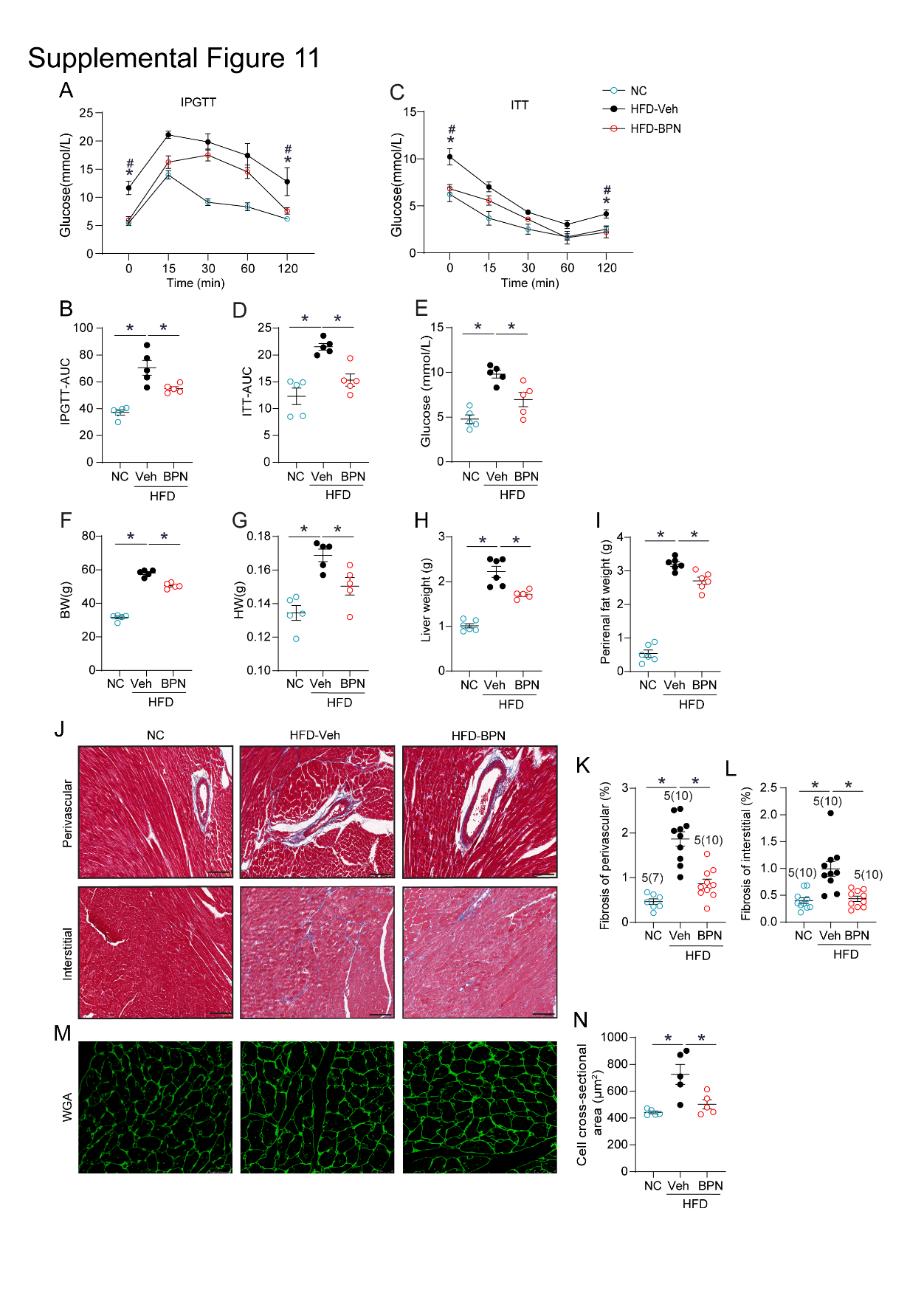


**Fig S11**. WT mice were fed with HFD for 4.5 months followed by treatment with saline or the PDE4D inhibitor BPN for 4 weeks. A-D) Mice were then subjected to IPGTT and IPITT tests. The time courses and AUC of blood glucose after intraperitoneal glucose and insulin injection were detected. body weight, heart weight, liver weight and visceral fat weight. E) The glucose levels were measured after 6 hrs of fasting. F-I) The body weight, heart, liver and fat weights were measured in mice after saline and BPN treatments. J-L) Cardiac fibrosis was detected by Masson's staining, and the perivascular and interstitial fibrosis were analyzed in saline and BPN-treated mice. Scale bar = 50 µm. M and N) Cardiac tissues were analyzed with WGA staining, and the myocyte cell sizes were analyzed.** p* < 0.05, HFD-Veh *vs* NC; *^#^ p* < 0.05, HFD-BPN *vs* HFD-Veh; by two-way ANOVA followed by Tukey’s test in panel A and C, and by one-way ANOVA followed by Tukey’s test in other panels.
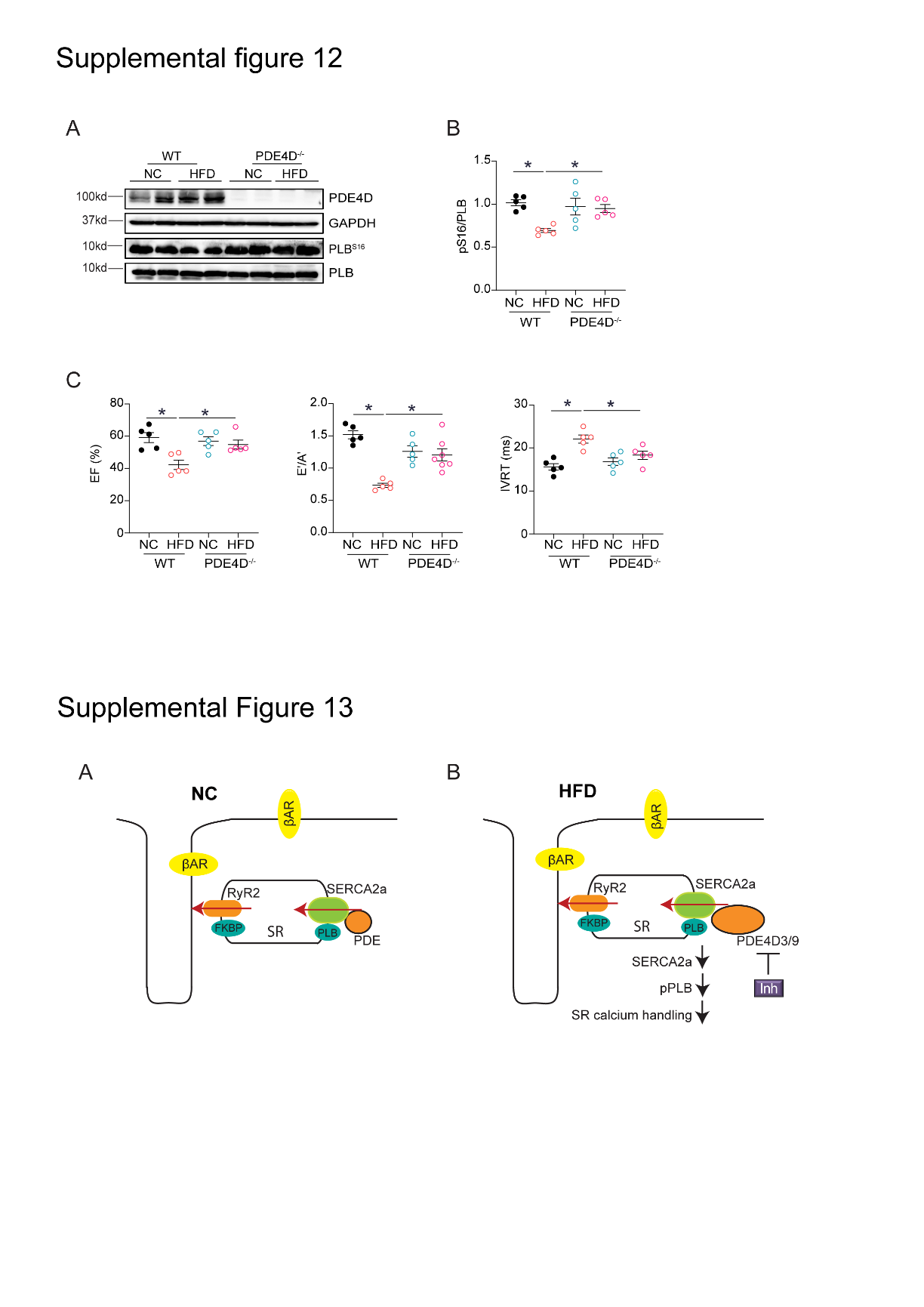


**Fig. S12** PDE4D knockout mice and WT littermates were fed with HFD diet for 4.5 months. A and B) Detection and quantification of PDE4D and PLB and phosphorylation of PLB at serine 16 in 2 month old WT and PDE4D knockout (PDE4D^-/-^) hearts. C) WT and PDE4D^-/-^ mice underwent NC and HFD for 4.5 months. The changes in diastolic and systolic function in WT and PDE4D^-/-^ measured and plotted. ** p* < 0.05 by two-way ANOVA followed by Tukey’s test.


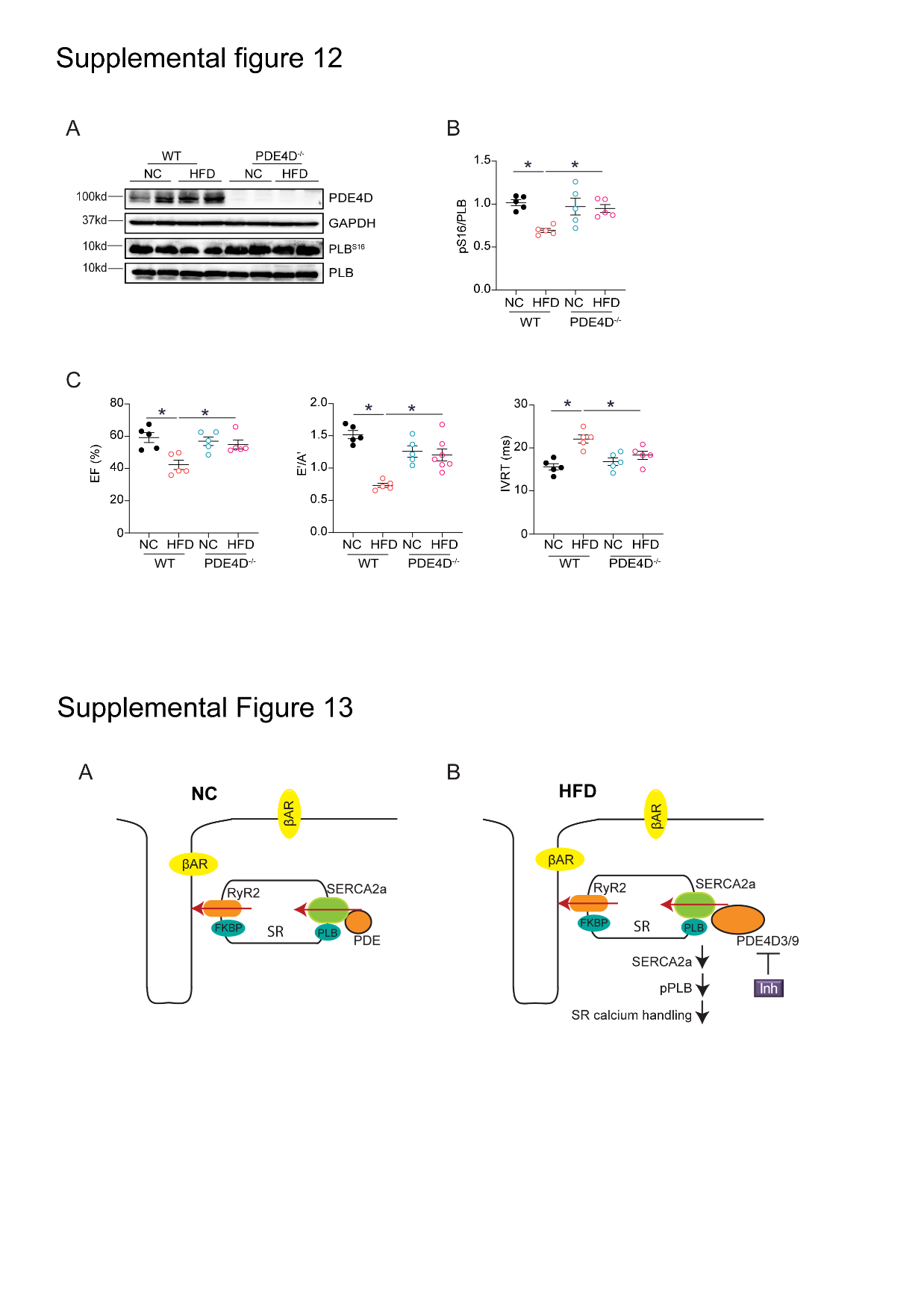


**Fig. S13**. **Schematic representation of the mechanism of SERCA2a regulation by PDE4D3/9 isoforms in DCM.** PDE4D3 and PDE4D9 are selectively upregulated in diabetic myocytes, where these isoforms specifically diminish the local PKA activity associated with SERCA2a but not RyR2 nanodomains. The higher levels of PDE4D3/9 are accompanied by reduced the levels of phosphorylation at the Ser 16 of PLB and calcium decay tau, contributing to suppressed SR calcium handling together with reduced calcium transient amplitude and contractile shortening. Inhibition of these PDE4D isoforms with membrane permeable dominant negative NT peptides restores PKA activity, phosphorylation of PLB, calcium handling, and contractility in diabetic myocytes.

**Table S1. WT NC and HFD feeding for 4 months** **(n=9, mean ± SEM).**

|  | NC | HFD |
| --- | --- | --- |
| HR (bpm) | 466.70±6.15 | 470.80±6.04 |
| IVS; d(mm) | 0.63±0.03 | 0.73±0.02* |
| IVS; s(mm) | 0.96±0.03 | 1.08±0.04 |
| LVID; d(mm) | 3.84±0.06 | 3.96±0.04 |
| LVID; s(mm) | 2.85±0.06 | 3.04±0.05* |
| LVPW; d(mm) | 0.72±0.04 | 0.96±0.03* |
| LVPW; s(mm) | 0.92±0.04 | 1.25±0.03* |
| EF % | 51.27±1.10 | 46.88±1.48* |
| FS % | 25.73±0.67 | 23.14±0.87* |
| LV Mass (mg) | 88.24±4.95 | 125.30±3.55* |
| Mass (corrected) | 70.59±3.96 | 100.30±2.84* |
| LV Vol; d(uL) | 63.75±2.51 | 68.48±1.70 |
| LV Vol; s(uL) | 31.13±1.57 | 36.37±1.58* |
| A' (mm/s) | -18.08±0.85 | -15.30±1.42 |
| E' (mm/s) | -19.07±0.84 | -13.63±1.53* |
| ET (ms) | 46.42±1.10 | 45.31±1.45 |
| IVCT (ms) | 17.69±1.08 | 21.03±1.94* |
| IVRT (ms) | 14.77±0.65 | 25.44±1.60* |
| A'/ E' | 0.95±0.05 | 1.20±0.14 |
| E'/ A' | 1.06±0.05 | 0.93±0.10 |
| MV A (mm/s) | 400.20±16.95 | 348.70±39.77 |
| MV E (mm/s) | 751.80±44.31 | 481.40±52.48* |
| MV E/A | 1.87±0.05 | 1.39±0.05* |
| MV E/E' | -40.17±3.53 | -37.27±5.36 |

^*^*P*<0.05, NC *versus* HFD.

|  | Vehicle  Pre-treat Post-treat | | Roflumilast  Pre-treat Post-treat | |
| --- | --- | --- | --- | --- |
| IVS; d (mm) | 0.8973±0.01865 | 0.8459±0.04183 | 0.9065±0.02752 | 0.7944±0.02626 |
| IVS; s (mm) | 1.135±0.03425 | 1.116±0.05171 | 1.122±0.03248 | 1.046±0.04039 |
| LVID; d (mm) | 3.921±0.1365 | 3.977±0.1230 | 3.581±0.1746 | 3.750±0.1165 |
| LVID; s (mm) | 3.088±0.1091 | 3.150±0.1224 | 2.854±0.1480 | 3.010±0.1004 |
| LVPW; d (mm) | 0.8827±0.04063 | 0.9399±0.01814 | 0.9839±0.02919 | 0.9126±0.04832 |
| LVPW; s (mm) | 1.001±0.04746 | 1.176±0.03179 | 1.093±0.01588 | 1.059±0.03916 |
| EF % | 43.76±0.6925 | 43.09±1.569 | 42.56±1.128 | 41.11±1.555 |
| FS % | 21.25±0.3913 | 20.93±0.8671 | 20.41±0.5921 | 19.73±0.8757 |
| LV Mass (mg) | 132.0±6.971 | 135.5±6.543 | 124.3±6.807 | 116.3±7.158 |
| Mass (corrected) | 105.6±5.577 | 108.4±5.234 | 99.44±5.446 | 93.02±5.726 |
| LV Vol; d (uL) | 67.49±5.417 | 69.65±4.884 | 55.54±6.219 | 60.81±4.692 |
| LV Vol; s (uL) | 38.00±3.178 | 39.99±3.472 | 32.21±3.886 | 35.87±3.054 |
| A' (mm/s) | -11.06±1.809 | -10.16±1.191 | -10.95±0.8708 | -11.58±0.8710 |
| E' (mm/s) | -10.39±1.019 | -8.610±1.265 | -9.715±0.7515 | -10.71±1.697 |
| ET (ms) | 43.10±0.9636 | 43.96±1.858 | 39.73±1.436 | 40.57±1.360 |
| IVCT (ms) | 23.71±1.678 | 25.15±2.107 | 24.01±0.6728 | 21.61±1.356* |
| IVRT (ms) | 26.34±1.541 | 27.47±1.842 | 25.75±0.9421 | 21.61±1.510* |
| A'/ E' | 1.062±0.1362 | 1.232±0.09189 | 1.194±0.1277 | 1.208±0.1664 |
| E'/ A' | 1.036±0.1497 | 0.8386±0.06073 | 0.9473±0.1212 | 0.9300±0.1309 |
| MV A (mm/s) | 258.1±20.42 | 319.5±37.17 | 320.6±31.95 | 296.5±33.00 |
| MV E (mm/s) | 469.0±52.66 | 498.4±86.88 | 430.6±45.04 | 479.7±61.01 |
| MV E/A | 1.803±0.1207 | 1.576±0.2125 | 1.378±0.1021 | 1.704±0.2297 |
| MV E/E' | -50.62±7.554 | -70.04±13.11 | -47.12±5.721 | -44.23±5.943 |

**Table** **S2. HFD before and after roflumilast treatment (n= 6, mean ± SEM).**

^*^*P*<0.05, roflumilast Post-treat *versus* roflumilast Pre-treat.

|  | Vehicle  Pre-treat Post-treat | | BPN14770  Pre-treat Post-treat | |
| --- | --- | --- | --- | --- |
| Diameter; d (mm) | 4.008±0.08749 | 3.846±0.2595 | 4.000±0.1327 | 3.7574±0.1325 |
| Diameter; s (mm) | 2.953±0.07090 | 2.894±0.1103 | 3.040±0.09629 | 2.602±0.1228* |
| Volume; d (mm) | 68.93±2.934 | 64.14±4.182 | 69.05±4.570 | 60.90±4.996 |
| Volume; s (mm) | 33.84±2.002 | 32.46±3.157 | 32.95±2.386 | 23.48±2.837* |
| LVPW; d (mm) | 0.8277±0.05210 | 0.8401±0.05209 | 0.9035±0.07018 | 0.7358±0.01532* |
| LVPW; s (mm) | 1.142±0.03970 | 1.075±0.03236 | 1.119±0.05013 | 1.187±0.03870 |
| EF % | 50.33±1.754 | 48.31±2.025 | 52.50±1.914 | 59.85±1.267* |
| FS % | 26.31±0.7413 | 24.80±1.334 | 26.22±1.748 | 31.69±1.294 * |
| LV Mass (mg) | 137.9±8.782 | 128.6±13.08 | 135.7±8.433 | 103.2±4.944* |
| Mass (corrected) | 110.3±6.746 | 102.9±10.46 | 108.6±6.746 | 82.57±3.955* |
| Stroke Volume | 35.09±1.806 | 31.68±1.869 | 36.10±3.345 | 37.41±3.281 |
| A' (mm/s) | -21.73±1.372 | -20.78±1.796 | -19.27±1.620 | -19.47±1.023 |
| E' (mm/s) | -17.06±1.161 | -15.07±1.214 | -14.33±1.029 | -23.49±1.956* |
| IVRT (ms) | 21.13±0.7543 | 22.37±0.8972 | 20.50±0.8319 | 15.92±0.3926* |
| A'/ E' | 1.315±0.05421 | 1.362±0.05421 | 1.256±0.03962 | 0.8310±0.03590* |
| E'/ A' | 0.7678±0.02912 | 0.7425±0.03013 | 0.8015±0.02441 | 1.1894±0.1334* |

**Table** **S3. HFD before and after BPN14770 treatment (n=6, mean ± SEM).**

^*^*P*<0.05, BPN14770 Post-treat *versus* BPN14770 Pre-treat.
